# APEX Nuclease (Multifunctional DNA Repair Enzyme) 1 Gene Asp148Glu Polymorphism and Cancer Risk: A Meta-Analysis Involving 58 Articles and 48903 Participants

**DOI:** 10.1371/journal.pone.0083527

**Published:** 2013-12-12

**Authors:** Dan Hu, Xiandong Lin, Hejun Zhang, Xiongwei Zheng, Wenquan Niu

**Affiliations:** 1 Department of Pathology, Fujian Provincial Tumor Hospital, Teaching Hospital of Fujian Medical University, Fuzhou, Fujian province, China; 2 Fujian Provincial Key Laboratory of Translational Center Medicine, Fujian Provincial Tumor Hospital, Teaching Hospital of Fujian Medical University, Fuzhou, Fujian province, China; 3 State Key Laboratory of Medical Genomics, Ruijin Hospital, School of Medicine, Shanghai Jiao Tong University, Shanghai, China; Duke-NUS, Singapore

## Abstract

**Background:**

Polymorphisms in the APEX nuclease (multifunctional DNA repair enzyme) 1 gene (*APEX1*) may be involved in the carcinogenesis by affecting DNA repair. We aimed to summarize available data on the association of the *APEX1* Asp148Glu (rs1130409) polymorphism with risk of multiple types of cancer via a meta-analysis.

**Methods and Results:**

In total, 58 qualified articles including 22,398 cancer patients and 26,505 controls were analyzed, and the data were extracted independently by two investigators. Analyses of the full data set indicated a marginally significant association of the *APEX1* Asp148Glu polymorphism with cancer risk under allelic (odds ratio (OR)=1.05; 95% confidence interval (95% CI): 0.99-1.11; P=0.071), dominant (OR=1.09; 95% CI: 1.01-1.17; P=0.028), and heterozygous genotypic (OR=1.08; 95% CI: 1.01-1.16; P=0.026) models, with significant heterogeneity and publication bias. In subgroup analyses by cancer type, with a Bonferroni corrected alpha of 0.05/6, significant association was observed for gastric cancer under both dominant (OR=1.74; 95% CI: 1.2-2.51; P=0.003) and heterozygous genotypic (OR=1.66; 95% CI: 1.2-2.31; P=0.002) models. In subgroup analysis by ethnicity, risk estimates were augmented in Caucasians, especially under dominant (OR=1.11; 95% CI: 1.0-1.24; P=0.049) and heterozygous genotypic (OR=1.11; 95% CI: 0.99-1.24; P=0.063) models. By study design, there were no significant differences between population-based and hospital-based studies. In subgroup analysis by sample size, risk estimates were remarkably overestimated in small studies, and no significance was reached in large studies except under the heterozygous genotypic model (OR=1.23; 95% CI: 1.06-1.43; P=0.006, significant at a Bonferroni corrected alpha of 0.05/2). By quality score, the risk estimates, albeit nonsignificant, were higher in low-quality studies than in high-quality studies. Further meta-regression analyses failed to identify any contributory confounders for the associated risk estimates.

**Conclusions:**

Our findings suggest that *APEX1* Asp148Glu polymorphism might be a genetic risk factor for the development of gastric cancer. Further investigations on large populations are warranted.

## Introduction

Polymorphisms in the APEX nuclease (multifunctional DNA repair enzyme) 1 gene (*APEX1*) may be involved in the carcinogenesis by correcting DNA damage [1]. The *APEX1* encodes the major apurinic/apyrimidinic endonuclease in human cells, and the loss of bases in apurinic/apyrimidinic sites can usually block the progress of the DNA replication apparatus and cause mutations. Therefore, the genetic defects responsible for the repair capacity of the *APEX1* are often regarded as the logical candidates for its functional investigations. It is worth noting that a single transition of the 1349^th^ base pair T allele to G allele, inducing the substitution of the 148^th^ amino acid aspartate (Asp) to glutamate (Glu) (Asp148Glu, rs1130409), in the 5^th^ exon of the *APEX1*, has been extensively investigated in association with a wide range of cancers, such as lung cancer, breast cancer, and bladder cancer [2-4]. The results of individual association studies in the literature, however, are often controversial and inconclusive. Taking lung cancer as an example, the *APEX1* 148Glu allele was a risk-conferring factor in Caucasians [5], but a risk-reducing factor in Asians [6]. As a caveat, this lack of consistency might be attributable to the presence of genetic heterogeneity across ethnic populations, the insufficient sample sizes involved, and the possibly uncontrolled confounding effects. To shed some light on these issues and to generate more information, we sought to summarize available data on the association of the *APEX1* Asp148Glu polymorphism with all types of cancers from both English and Chinese literature via a meta-analysis, and further to explore the potential sources of between-study heterogeneity and the possible existence of publication bias.

## Methods

Meta-analysis of observational studies poses particular challenges owing to its inherent biases and divergences in study design. We therefore carried out this meta-analysis according to the guidelines set forth by the Meta-analysis Of Observational Studies in Epidemiology (MOOSE) statement [7] (Please see the Checklist S1).

### Search strategy

Four databases including the PubMed, EMBASE (Excerpta Medica database), Wanfang (http://www.wanfangdata.com.cn), and CNKI (China National Knowledge Infrastructure, http://www.cnki.net) were searched on May 1, 2013 for observational studies investigating the association between the *APEX1* Asp148Glu polymorphism and all types of cancers. Subject terms used for the search were: ‘apurinic/apyrimidinic’, ‘APE1’, ‘*APEX1*’, ‘cancer’, ‘tumor’, ‘neoplasm’, combined with ‘gene’, ‘polymorphism’, ‘variant’, ‘mutation’, ‘allele’, or ‘genotype’. The reference lists of all the retrieved articles as well as those of reviews on the same topic were also searched to identify the additional missing articles. Searching results were limited to studies with a case-control design and articles published in the English or Chinese language.

### Study selection

Two investigators (Dan Hu and Wenquan Niu) independently obtained the full texts of potentially eligible articles on the basis of their titles and abstracts. To avoid the double counting of the participants recruited in more than one publication, article authors were emailed for inquiry when necessary. In case of more than one publication from the same study population, the data from the most recent or the most complete publication were extracted.

### Inclusion/exclusion criteria

Our analyses were limited to the studies that strictly fulfilled the following inclusion criteria (all points must be satisfied for inclusion): (1) clinical endpoint (dependent variable): all types of cancers; (2) study design: either retrospective or nested case-control design; (3) independent variables: the genotype and/or allele counts of the *APEX1* Asp148Glu polymorphism. Studies were excluded (one point was sufficient for exclusion) if they investigated the progression, severity, phenotype modification, and the response to treatment or survival, as well as if they were conference abstracts, case reports or series, editorials, narrative reviews, and the non-English and non-Chinese articles.

### Data extraction

The data were extracted from all the qualified articles independently by two investigators (Dan Hu and Wenquan Niu) according to a standardized Excel template (Microsoft Corp, Redmond, WA). The discrepancies were resolved by the discussion and review of original articles, and a consensus was reached finally.

The data were collected on the first author, year of publication, ethnicity of the study population, cancer type, study design, case-control status, the genotypes/alleles of the *APEX1* Asp148Glu polymorphism between patients and controls, and the demographic data, if available, including age, gender, smoking, and drinking.

### Quality score assessment

The study quality was evaluated by using a quality assessment score developed for genetic association studies by Thakkinstian and colleagues [8]. Total scores range from 0 (the worst) to 12 (the best). The criteria for quality assessment of genetic associations between the *APEX1* Asp148Glu polymorphism and cancer are described in the Table S1.

### Statistical analyses

In this meta-analysis, four genetic models of inheritance were performed for *APEX1* Asp148Glu polymorphism including allelic model (the 148Glu allele versus the 148Asp allele), dominant model (the 148Glu/148Glu genotype plus the 148Glu/Asp genotype versus the 148Asp/Asp genotype), homozygous (the 148Glu/148Glu genotype versus the 148Asp/Asp genotype) and heterozygous (the 148Glu/Asp genotype versus the 148Asp/Asp genotype) genotypic models.

The random-effects model using the DerSimonian & Laird method was employed to compute the weighted odds ratios (ORs) and the corresponding 95% confidence intervals (95% CIs). Heterogeneity between studies was evaluated by the χ^2^ test, and was quantified by the inconsistency index (*I* ^2^) statistic, which ranges from 0% to 100% and is defined as the percentage of the observed between-study variability that is due to heterogeneity rather than chance.

Predefined subgroup analyses were performed a priori according to the cancer type, ethnicity of the study populations (Caucasian, Asian, African-American, or mixed), study design (population-based or hospital-based), the total sample size (<300 subjects or ≥300 subjects), and the quality score (score <7 or score ≥7). For a certain cancer, the data were presented and summarized if there were three or more independent studies that provided the genotype or allele counts of the Asp148Glu polymorphism between patients and controls.

Meta-regression analyses were performed to estimate the extent to which different study-level variables, including age, smoking, drinking, and quality score, explained the potential heterogeneity of pooled effect estimates of the *APEX1* Asp148Glu polymorphism on cancer risk.

Besides the Egger’s test, publication bias was evaluated by the trim-and-fill method, which can estimate the number and outcomes of theoretically missing studies due to publication bias. P<0.05 was considered statistical significance, except for the *I* ^2^ and Egger’s statistics, for which significance was defined as P<0.10 [9]. All statistical analyses were conducted by the STATA software (StataCorp, TX, version 11.2 for Windows).

## Results

### Eligible articles

**Figure 1:**
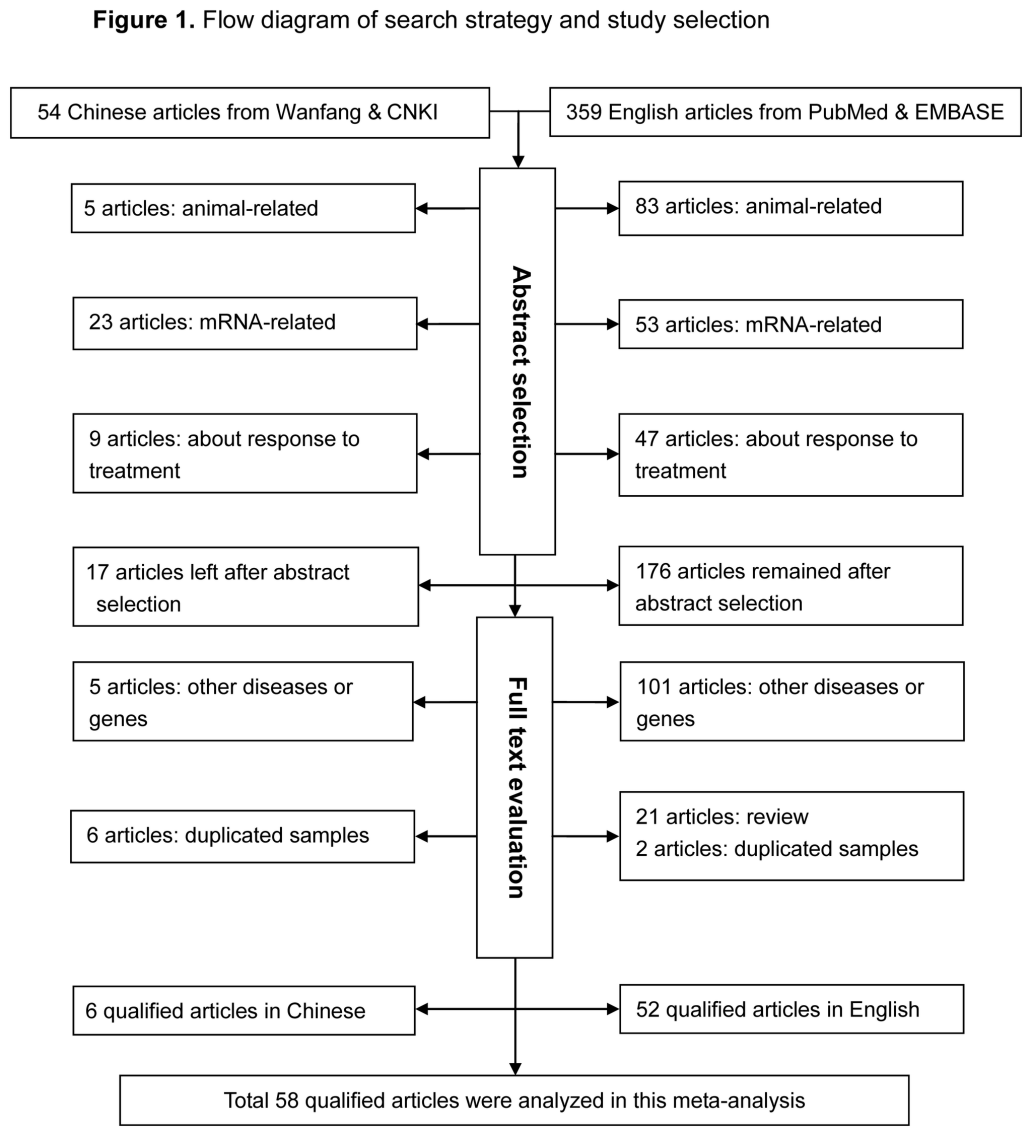
Flow diagram of search strategy and study selection.

A flow diagram schematizing the process of article selection with specific reasons is presented in Figure 1. In total, 413 potentially relevant articles were identified after the initial search, and 58 of them were deemed as eligible after applying further inclusion/exclusion criteria [3-6,10-63]. All qualified articles, including 52 articles written in English and 6 articles in Chinese [39,48,51,52,55,57], were published between the year 2003 and 2013. Because five articles provided data by ethnicity, two by cancer type, and two by the presence of menopause, there were 68 independent populations for comparisons in final analyses.

### Study characteristics

The baseline characteristics of all qualified populations are shown in Table 1, and the genotype distributions and allele frequencies of the *APEX1* Asp148Glu polymorphism between cancer patients and controls of all qualified populations are presented in the Table S2. Of 68 qualified populations, 14 were conducted for lung cancer, 10 for colorectal cancer, 9 for bladder cancer, 8 for breast cancer, 6 for prostate cancer, 4 for gastric cancer, 2 for pancreatic cancer, 2 for head and neck cancer, 2 for leukaemia cancer, and 1 for melanoma, biliary tract, cervical, esophageal, thyroid, hepatocellular, gioma, cervical, renal, endometrical carcinoma, and prostate cancers, respectively. The quality scores of all 68 populations ranged from 3 to 12, with a mean value of 6.9 (standard deviation: 1.92). Moreover, there were 30 populations involving Caucasians, 29 involving Asians, 4 involving African-Americans, and 5 involving the mixed populations. There were 27 populations conducted on a population-based design and 41 on a hospital-based design. 32 of 68 populations (47.1%) had the total sample size (the sum of patients and controls) equal to or greater than 300 participants in this meta-analysis.

**Table 1:** The baseline characteristics of the study populations analyzed in this meta-analysis.

**First author (year)**	**Quality score**	**Cancer type**	**Ethnicity**	**Design**	**Sample size**		**Age (years)**	
					**Cases**	**Controls**	**Cases**	**Controls**
Misra RR et al (2003)	5	Lung	Caucasian	Population	315	315	60	59
Popanda O et al (2004)	7	Lung	Caucasian	Hospital	463	460	61	55
Ito H et al (2004)	9	Lung	Asian	Hospital	178	449	62.9	62.6
Chen L et al (2005)	6	Prostate	African-Americans	Population	124	116	64	59
Chen L et al (2005)	6	Prostate	Caucasian	Population	228	219	64	62
Shen M et al (2005)	5	Lung	Asian	Population	119	113	55	55
Broberg K et al (2005)	6	Bladder	Caucasian	Population	63	158	69	69
Zienolddiny S et al (2006)	9	Lung	Caucasian	Population	343	413	65	60
Zhang Y et al (2006) (Postmenopausal)	7	Breast	Caucasian	Population	839	679	NA	NA
Zhang Y et al (2006) (Premenopausal)	7	Breast	Caucasian	Population	587	434	NA	NA
Terry PD et al (2006)	6	Bladder	Mixed	Hospital	239	215	65.7	63.3
Moreno V et al (2006)	10	Colorectal	Caucasian	Hospital	359	312	NA	NA
Li C et al (2006)	6	Melanoma	Caucasian	Hospital	602	603	NA	NA
Li J et al (2006)	6	Pancreatic	Mixed	Hospital	384	357	NA	NA
Li C et al (2007)	6	Head and neck	Caucasian	Hospital	830	854	NA	NA
Huang M et al (2007)	5	Bladder	Caucasian	Hospital	596	590	63.94	62.77
Figueroa JD et al (2007)	7	Bladder	Caucasian	Hospital	1150	1149	66	65
De Ruyck K et al (2007)	6	Lung	Caucasian	Hospital	110	110	62	61
Berndt S et al (2007)	11	Colorectal	Mixed	Population	767	773	NA	NA
Berndt S et al (2007)	11	Colorectal	Caucasian	Population	720	725	NA	NA
Chang JS et al (2008)	5	Lung	Mixed	Population	113	299	65.85	66.3
Chang JS et al (2008)	5	Lung	African-Americans	Population	255	280	63.51	61.81
Zhu R et al (2008)	5	Leukaemia	Asian	Hospital	105	108	NA	NA
Tse D et al (2008)	8	Esophageal	Caucasian	Hospital	312	454	64	64
Smith TR et al (2008)	7	Breast	Caucasian	Hospital	336	416	57.4	58.7
Smith TR et al (2008)	7	Breast	African-Americans	Hospital	63	78	57.4	58.7
Shekari M et al (2008)	6	Cervical	Asian	Hospital	138	180	48.55	48.81
Pardini B et al (2008)	7	Colorectal	Caucasian	Hospital	532	532	58.5	57.4
Mitra AK et al (2008)	5	Breast	Asian	Population	155	235	NA	NA
Kasahara M et al (2008)	6	Colorectal	Asian	Hospital	68	121	67.3	67.4
Huang WY et al (2008)	7	Biliary tract	Asian	Population	411	786	NA	NA
Chiang FY et al (2008)	7	Thyroid	Asian	Hospital	283	469	45.3	43.9
Andrew AS et al (2008)	8	Bladder	Caucasian	Hospital	1029	1281	NA	NA
Sangrajrang S et al (2008) (Postmenopausal)	9	Breast	Asian	Hospital	239	180	48	45.3
Sangrajrang S et al (2008) (Premenopausal)	9	Breast	Asian	Hospital	268	245	48	45.3
Narter KF et al (2009)	4	Bladder	Caucasian	Hospital	83	45	63.43	59.98
Lu J et al (2009)	9	Lung	Asian	Population	500	517	NA	NA
Lo YL et al (2009)	7	Lung	Asian	Hospital	730	730	60.77	60.8
Liu Y et al (2009)	7	Glioma	Caucasian	Population	373	365	NA	NA
Gangwar R et al (2009)	7	Bladder	Asian	Hospital	206	250	59	57.8
Agachan B et al (2009)	3	Lung	Caucasian	Hospital	98	67	51.26	48.81
Ji L et al (2009)	4	Hepatocellular	Asian	Hospital	500	507	NA	NA
Ye CC et al (2010)	6	Colorectal	Asian	Hospital	123	158	60.9	NA
Wang M et al (2010)	6	Bladder	Asian	Hospital	234	253	63.5	62.9
Palli D et al (2010)	9	Gastric	Caucasian	Population	314	548	68.8	55.5
Osawa K et al (2010)	6	Lung	Asian	Hospital	104	120	66.3	67.3
Jelonek K et al (2010)	5	Colorectal	Caucasian	Hospital	103	153	NA	NA
Jelonek K et al (2010)	5	Head and neck	Caucasian	Hospital	104	110	NA	NA
Jelonek K et al (2010)	5	Breast	Caucasian	Hospital	91	412	NA	NA
Brevik A et al (2010)	5	Colorectal	Caucasian	Population	304	359	NA	NA
Canbay E et al (2010)	7	Gastric	Caucasian	Population	50	247	60.07	52.8
Agalliu I et al (2010)	9	Prostate	Caucasian	Population	1308	1266	NA	NA
Agalliu I et al (2010)	9	Prostate	African-Americans	Population	149	85	NA	NA
Wang MM et al (2010)	6	Cervical	Asian	Hospital	306	306	46.84	46.04
Huang LZ et al (2011)	6	Leukaemia	Asian	Hospital	415	519	NA	NA
Li Z et al (2011)	10	Lung	Asian	Hospital	455	443	59.68	58.39
Kuasne H et al (2011)	4	Prostate	Mixed	Hospital	172	172	65.64	63.86
Gu D et al (2011)	7	Gastric	Asian	Hospital	338	362	61.76	62.46
Cao Q et al (2011)	6	Renal	Asian	Hospital	612	632	56.9	56.7
Canbay E et al (2011)	9	Colorectal	Caucasian	Population	79	247	60.22	59.73
Deng Q et al (2011)	4	Lung	Asian	Population	315	315	59	58
Zhonghua L et al (2011)	5	Gastric	Asian	Hospital	126	156	58.7	53.1
Nakao M et al (2012)	9	Pancreatic	Asian	Population	185	1465	NA	NA
Mittal RD et al (2012)	9	Prostate	Asian	Population	195	250	66	64.7
Mittal RD et al (2012)	9	Bladder	Asian	Population	212	250	NA	NA
Mandal R et al (2012)	12	Prostate	Asian	Population	192	224	62.6	59.1
Cincin Z et al (2012)	4	Endometrial carcinoma	Caucasian	Hospital	104	158	56.2	53.71
Li Y et al (2013)	6	Colorectal	Asian	Hospital	451	631	59.4	57

Abbreviations: NA, not available.

### Overall analyses

**Figure 2:**
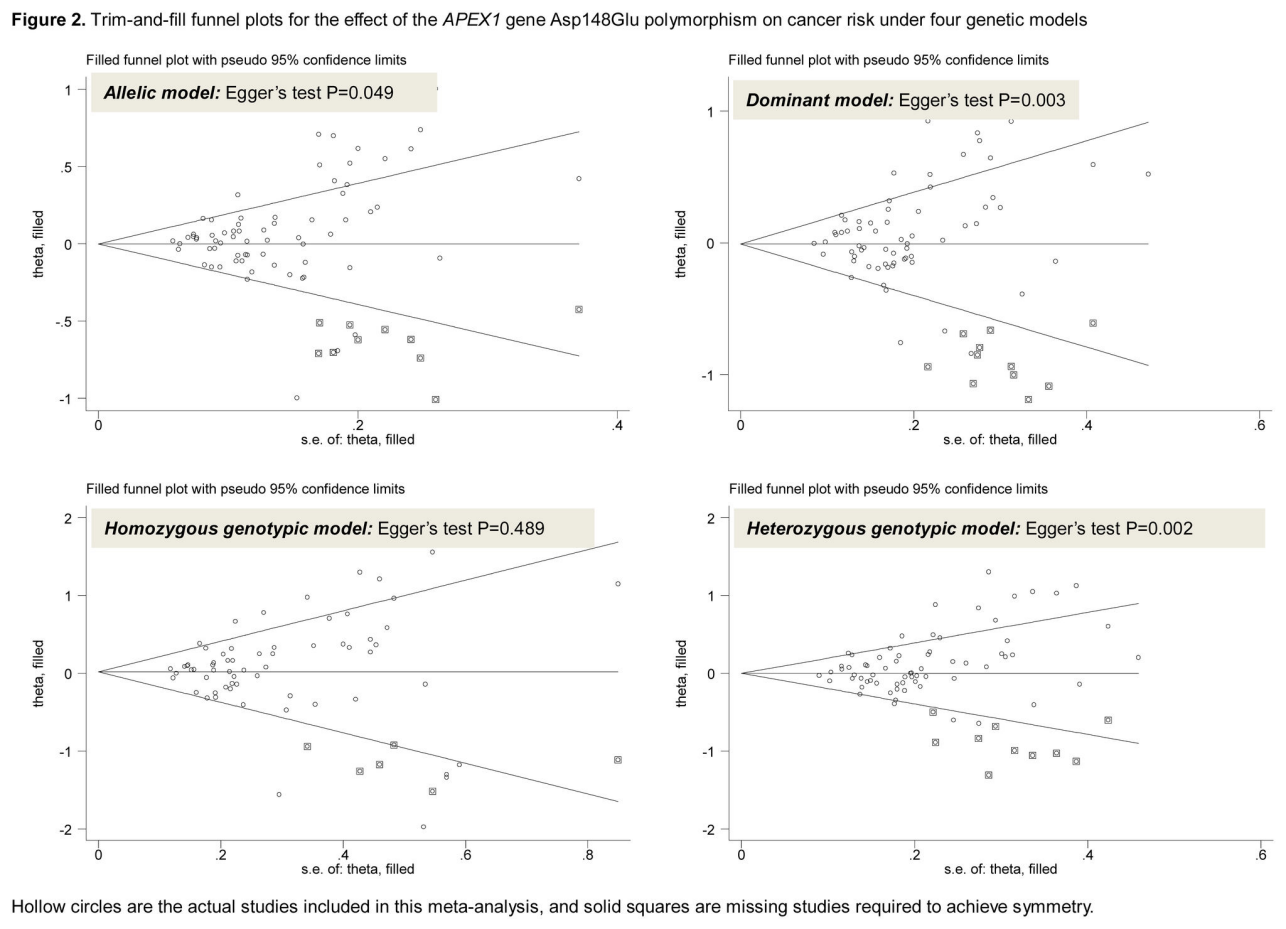
Trim-and-ﬁll funnel plots for the effect of the *APEX1* Asp148Glu polymorphism on cancer risk under four genetic models. Hollow circles are the actual studies included in this meta-analysis, and solid squares are missing studies required to achieve symmetry.

Analyses of the full data set indicated a marginally significant association of the *APEX1* Asp148Glu polymorphism with cancer risk under allelic (OR=1.05; 95% CI: 0.99-1.11; P=0.071), dominant (OR=1.09; 95% CI: 1.01-1.17; P=0.028), and heterozygous genotypic (OR=1.08; 95% CI: 1.01-1.16; P=0.026) models, with high probabilities of heterogeneity (*I* ^2^=70.6%, 67.1%, and 59.5% respectively, all P<0.0005 from the χ^2^ test) (Table 2 and Table 3). Moreover, the probability of publication bias was high as reflected by both the Egger’s tests and the trim-and-ﬁll funnel plots for these three models (Figure 2). We estimated that there were respectively 10, 11, and 10 missing independent populations to make the funnel plots symmetrical under allelic, dominant, and heterozygous genotypic models.

**Table 2:** Overall and subgroup estimates of the associations of *APEX1* Asp148Glu polymorphism with cancer risk under allelic and dominant models.

**Groups/subgroups**	**Number of studies (cases/controls)**	**Allelic model**			**Dominant model**		
		**OR; 95% CI; P**	***I*^2^ (P)**	**P_Egger_**	**OR; 95% CI; P**	***I*^2^ (P)**	**P_Egger_**
**Overall estimates**	68 (22398/26505)	1.05; 0.99-1.11; 0.071	70.6% (<0.0005)	0.049	1.09; 1.01-1.17; 0.028	67.1% (<0.0005)	0.003
**Cancer type**							
Lung cancer	14 (4007/4513)	1.06; 0.95-1.19; 0.325	66.8% (<0.0005)	0.018	1.1; 0.93-1.3; 0.268	67.6% (<0.0005)	0.01
Colorectal cancer	10 (3459/3978)	1.07; 0.94-1.22; 0.325	72.2% (<0.0005)	0.814	1.2; 0.97-1.49; 0.101	75.2% (<0.0005)	0.681
Bladder cancer	9 (3618/3918)	0.99; 0.92-1.06; 0.701	3.4% (0.406)	0.481	0.99; 0.89-1.11; 0.903	10.4% (0.348)	0.058
Breast cancer	8 (2546/2655)	1.03; 0.88-1.21; 0.695	69.3% (0.002)	0.68	1.05; 0.82-1.34; 0.704	71.8% (0.001)	0.681
Prostate cancer	6 (2122/2046)	1.08; 0.98-1.2; 0.11	5.7% (0.38)	0.103	1.13; 0.95-1.35; 0.172	28.9% (0.218)	0.191
Gastric cancer	4 (803/1311)	1.42; 1.09-1.84; 0.009	71.0% (0.016)	0.16	1.74; 1.2-2.51; 0.003	64.9% (0.036)	0.082
**Ethnicity**							
Caucasian	30 (12044/13249)	1.06; 0.99-1.13; 0.116	66.5% (<0.0005)	0.022	1.11; 1.0-1.24; 0.049	67.8% (<0.0005)	0.011
Asian	29 (8161/10945)	1.03; 0.64-1.14; 0.508	78.8% (<0.0005)	0.617	1.05; 0.93-1.19; 0.438	71.6% (<0.0005)	0.076
African-American	4 (573/546)	1.03; 0.86-1.22; 0.762	0.0% (0.578)	0.56	0.98; 0.77-1.25; 0.868	0.0% (0.507)	0.461
Mixed	5 (1620/1765)	1.07; 0.92-1.23; 0.375	44.1% (0.128)	0.637	1.2; 0.95-1.53; 0.132	54.2% (0.068)	0.802
**Study design**							
Population-based	27 (8984/11489)	1.04; 0.97-1.11; 0.255	53.7% (0.001)	0.054	1.10; 0.99-1.22; 0.085	60.9% (<0.0005)	0.035
Hospital-based	41 (13414/15016)	1.05; 0.98-1.14; 0.187	76.7% (<0.0005)	0.25	1.08; 0.97-1.19; 0.148	70.8% (<0.0005)	0.039
**Sample size**							
≥300 participants	32 (17084/18154)	0.99; 0.94-1.04; 0.667	63.2% (<0.0005)	0.071	0.99; 0.93-1.06; 0.834	50.2% (0.001)	0.509
<300 participants	36 (5314/8351)	1.16; 1.05-1.3; 0.006	73.5% (<0.0005)	0.016	1.26; 1.08-1.47; 0.003	73.1% (<0.0005)	0.003
**Quality score**							
≥7	34 (13846/16752)	1.03; 0.98-1.08; 0.238	46.0% (0.0085)	0.202	1.06; 0.98-1.14; 0.152	49.1% (0.001)	0.061
<7	(8477/9718)	1.07; 0.97-1.19; 0.175	80.7% (<0.0005)	0.143	1.13; 0.98-1.3; 0.099	76.8% (<0.0005)	0.019

Abbreviations: OR, odds ratio; 95% CI, 95% confidence interval.

**Table 3:** Overall and subgroup estimates of the associations of *APEX1* Asp148Glu polymorphism with cancer risk under two genotypic models.

**Groups/subgroups**	**Homozygous genotypic model**			**Heterozygous genotypic model**		
	**OR; 95% CI; P**	***I*^2^ (P)**	**P_Egger_**	**OR; 95% CI; P**	***I*^2^ (P)**	**P_Egger_**
**Overall estimates**	1.06; 0.96-1.17; 0.236	62.5% (<0.0005)	0.489	1.08; 1.01-1.16; 0.026	59.5% (<0.0005)	0.002
**Cancer type**						
Lung cancer	1.07; 0.87-1.3; 0.537	54.9% (0.009)	0.058	1.11; 0.93-1.32; 0.26	65.9% (<0.0005)	0.008
Colorectal cancer	1.03; 0.8-1.33; 0.815	65.1 % (0.005)	0.158	1.25; 1.0-1.56; 0.055	74.7% (<0.0005)	0.529
Bladder cancer	0.94; 0.71-1.26; 0.686	56.5% (0.032)	0.482	1.0; 0.9-1.11; 0.974	3.3% (0.404)	0.045
Breast cancer	1.0; 0.78-1.27; 0.967	43.9% (0.086)	0.687	1.05; 0.82-1.34; 0.697	67.9% (0.003)	0.703
Prostate cancer	1.15; 0.95-1.4; 0.148	0.0% (0.705)	0.001	1.1; 0.91-1.33; 0.591	29.4% (0.214)	0.271
Gastric cancer	1.79; 1.11-2.89; 0.017	64.2% (0.039)	0.332	1.66; 1.2-2.31; 0.002	50.7% (0.107)	0.054
**Ethnicity**						
Caucasian	1.06; 0.94-1.2; 0.332	54.5% (<0.0005)	0.213	1.11; 0.99-1.24; 0.063	65.1% (<0.0005)	0.014
Asian	1.04; 0.85-1.27; 0.723	74.7% (<0.0005)	0.646	1.05; 0.94-1.17; 0.396	58.1% (<0.0005)	0.033
African-American	1.11; 0.77-1.61; 0.573	0.0% (0.71)	0.533	0.94; 0.73-1.22; 0.646	0.0% (0.554)	0.421
Mixed	1.05; 0.81-1.36; 0.724	21.2% (0.28)	0.708	1.24; 0.97-1.58; 0.083	52.1% (0.08)	0.83
**Study design**						
Population-based	1.03; 0.92-1.16; 0.571	33.2% (0.052)	0.151	1.12; 1.0-1.26; 0.051	63.2% (<0.0005)	0.025
Hospital-based	1.06; 0.92-1.23; 0.426	71.9% (<0.0005)	0.98	1.06; 0.97-1.16; 0.215	57.1% (<0.0005)	0.043
**Sample size**						
≥300 participants	1.21; 0.98-1.51; 0.082	64.6% (<0.0005)	0.164	1.23; 1.06-1.43; 0.006	69.1% (<0.0005)	0.812
<300 participants	0.99; 0.9-1.09; 0.849	57.3% (<0.0005)	0.918	1.01; 0.95-1.07; 0.797	31.1% (0.05)	0.005
**Quality score**						
≥7	1.05; 0.95-1.16; 0.317	43.5% (0.005)	0.736	1.06; 0.98-1.15; 0.131	50.8% (<0.0005)	0.056
<7	1.08; 0.89-1.32; 0.433	73.6% (<0.0005)	0.536	1.12; 0.98-1.27; 0.087	66.6% (<0.0005)	0.011

Abbreviations: OR, odds ratio; 95% CI, 95% confidence interval.

### Subgroup analyses

To account for the potential sources of between-study heterogeneity, a set of predefined subgroup analyses were conducted (Table 2, Table 3, and Figures S1-S5).

By cancer type, after the Bonferroni correction for the multiple testing (Bonferroni significance threshold P=0.05 divided by the number of cancers (n=6): P=0.0083), significant association was observed for gastric cancer under both dominant (OR=1.74; 95% CI: 1.2-2.51; P=0.003) and heterozygous genotypic (OR=1.66; 95% CI: 1.2-2.31; P=0.002) models, whereas no significance was reached for the other cancers under investigation. The heterogeneity between studies was relatively low for bladder and prostate cancers.

By ethnicity, the magnitude of risk estimates was marginally significant in Caucasians under both dominant (OR=1.11; 95% CI: 1.0-1.24; P=0.049) and heterozygous genotypic (OR=1.11; 95% CI: 0.99-1.24; P=0.063) models, whereas this significance failed to survive the stringent Bonferroni correction (Bonferroni significance threshold P=0.05 divided by the number of ethnicities (n=4): P=0.0125). In Asians and African-Americans, there was no significant association observed in this meta-analysis.

By study design, there were no significant differences in the pooled risk estimates between the population-based and hospital-based studies, with high probabilities of between-study heterogeneity and publication bias.

By sample size, the risk estimates were significantly overestimated in small studies (the total sample size <300 participants), and no significance was reached in large studies (the total sample size ≥300 participants) under all but heterozygous genotypic model (OR=1.23; 95% CI: 1.06-1.43; P=0.006), even after the Bonferroni correction (Bonferroni significance threshold P=0.05 divided by the number of 2 groups: P=0.025). There was moderate evidence of heterogeneity.

By quality score, the risk estimates were relatively higher in low-quality studies (quality score <7) than in high-quality studies (quality score ≥7), and there was no significance observed under all four genetic models. The presence of heterogeneity was more evident in low-quality studies than in high-quality studies. Significant publication bias was found under both dominant and heterozygous genotypic models.

### Meta-regression analyses

To further explore additional sources of between-study heterogeneity, we constructed a multivariable meta-regression model that included age, smoking, drinking, and quality score as independent variables. However, none of these variables were observed to significantly affect the relationship between the *APEX1* Asp148Glu polymorphism and cancer susceptibility.

## Discussion

Via a meta-analysis of the data from 58 articles and on 48903 participants, we investigated the association of the non-synonymous polymorphism Asp148Glu in *APEX1* with cancer risk. The principle finding of this study was that the *APEX1* 148Glu allele was associated with the significant risk of developing gastric cancer under both dominant and heterozygous genotypic models, even after the Bonferroni correction. Moreover, our subgroup analyses indicated that ethnicity might be an underlying cause of heterogeneity between studies. Although other sources of heterogeneity cannot be easily ruled out, this study, to the best of our knowledge, is so far the largest meta-analysis examining the association of the *APEX1* Asp148Glu polymorphism with cancer risk.

Recently, Zhou and colleagues have synthesized data from 32 case-control articles on the two polymorphisms of *APEX1*, and they failed to find any relationship between cancer risk and the Asp148Glu polymorphism [64]. By contrast, the findings of this meta-analysis supported the significant roles of the 148Glu allele in susceptibility to gastric cancer. However, a note of caution should be added because the risk estimates for gastric cancer were based on 803 patients and 1311 controls from 4 independent populations in this meta-analysis, the sample size might not be sufficient enough to derive a firm conclusion. It is recommended that to generate robust data, a much larger sample set encompassing more than 1000 participants in each group might be required [65]. A large, well-designed study is therefore warranted to confirm or refute the significance of our findings.

Moreover, extending the findings of the meta-analysis by Zhou and colleagues [64], we, in subgroup analyses, observed a marginally significant association of the *APEX1* Asp148Glu polymorphism with cancer risk in Caucasians under both dominant and heterozygous genotypic models, but not in Asians and African-Americans. One possible explanation for this divergence is the genetic heterogeneity across ethnicities. For example in this meta-analysis, the average frequency of the *APEX1* 148Glu allele was 34.82% in Asian controls, but was as exceedingly high as 45.21% in Caucasian controls. In general, genetic heterogeneity is an inevitable problem in any disease identification strategy. This ethnicity-specific effect suggests that different genetic backgrounds may account for this discrepancy or that different populations may have different linkage disequilibrium patterns due to the evolutionary history. As such, it is necessary to construct a database of susceptible genes and polymorphisms implicated in carcinogenesis in each ethnic group.

To seek additional sources of heterogeneity, an alternative method is to perform a meta-regression analysis; however, none of the confounders under study contributed remarkably to the presence of heterogeneity in this meta-analysis. It is important to bear in mind that meta-regression analysis, albeit enabling quantitative covariates to be considered, does not have the methodological rigor of a properly designed study that is intended to test the effect of these covariates formally. Admittedly, one limitation facing this method was the number of available studies with detailed information such as smoking and drinking. In fact, most studies did not report the study-level covariates of interest, precluding a more robust assessment of additional sources of heterogeneity.

Some limitations need to be acknowledged for this meta-analysis. First, all qualified studies were conducted on case-control design, which precludes further comments on a cause-effect relationship. Second, in both overall and subgroup analyses, most resultant associations might be biased by the moderate to high degree of between-study heterogeneity, which enhances the difficulty in drawing firm conclusions and encourages the exploration of other possible reasons for heterogeneity. Third, the overall findings of this study were skewed by publication bias, although publication bias was improved in most subgroups, possibly due to the lack of power for small number of studies involved. Factually as suggested by Hannah and colleagues, the study power is low if the number of studies included in a meta-analysis is 10 or fewer [66]. Moreover, potential selection bias cannot be completely ruled out, because we only retrieved studies from English and Chinese journals and published articles. Fourth, due to the relatively small sample sizes involved in subgroup analyses, we must hold some reservations about the interpretation of our subgroup results. Last but not the least, we only focused on the *APEX1* Asp148Glu polymorphism, and did not cover the other polymorphisms of *APEX1*. It is possible that the potential role of the examined polymorphism is diluted or masked by other gene-gene or gene-environment interactions. Thus, we cannot just to a conclusion until further confirmation of our findings has been undertaken.

In conclusion, via a meta-analysis of the data from 58 articles and on 48903 participants, we provide evidence that the *APEX1* Asp148Glu polymorphism might be a genetic risk factor for the development of gastric cancer. Nevertheless, despite the small sample sizes involved in subgroup analyses, this meta-analysis provides an anchoring point for better understanding of the pathogenesis of cancers. For practical reasons, we hope that this study will not remain just another endpoint of research instead of a beginning to establish the background data to understand the roles of the *APEX1* in carcinogenesis.

## Supporting Information

Table S1 **Criteria for quality assessment of genetic associations of the *APEX1* Asp148Glu polymorphism with cancer risk.**
(DOC)

Table S2 **The genotype distributions and allele frequencies of the *APEX1* Asp148Glu polymorphism between cancer patients and controls of all examined populations in this meta-analysis.**
(DOC)

Figure S1 **Forest plots of the lung, bladder, colorectal cancer (the upper panel), and prostate, breast, gastric cancers (the lower panel) in subgroup analyses by cancer type for the *APEX1* Asp148Glu polymorphism under the allelic model.**
(PDF)

Figure S2 **Forest plots of the Caucasians (the upper panel), Asians (the middle panel), African-Americans and mixed populations (the lower panel) in subgroup analyses by ethnicity for the *APEX1* Asp148Glu polymorphism under the allelic model.**
(PDF)

Figure S3 **Forest plots of the hospital-based studies (the upper panel), and population-based studies (the lower panel) in subgroup analyses by study design for the *APEX1* Asp148Glu polymorphism under the allelic model.**
(PDF)

Figure S4 **Forest plots of the small studies (the upper panel), and large studies (the lower panel) in subgroup analyses by sample size for the *APEX1* Asp148Glu polymorphism under the allelic model.**
(PDF)

Figure S5 **Forest plots of the high-quality studies (the upper panel), and low-quality studies (the lower panel) in subgroup analyses by sample size for the *APEX1* Asp148Glu polymorphism under the allelic model.**
(PDF)

Checklist S1 **MOOSE checklist.**
(DOC)
