# Supplementary material for: APEX Nuclease (Multifunctional DNA Repair Enzyme) 1 Gene Asp148Glu Polymorphism and Cancer Risk: A Meta-Analysis Involving 58 Articles and 48903 Participants
Source: PLoS One. 2013 Dec 12;8(12):e83527. doi: 10.1371/journal.pone.0083527 (PMC3861501; doi:10.1371/journal.pone.0083527)
Supplement: Table S1 — Criteria for quality assessment of genetic associations of the APEX1 Asp148Glu polymorphism with cancer risk. (DOC) [file pone.0083527.s001.doc]

**Supplementary Table S1. Criteria for quality assessment of genetic associations of the *APEX1* Asp148Glu polymorphism with cancer risk**

| ***Criteria*** | Quality score |
| --- | --- |
| ***Representativeness of cases*** | |
| 1. Consecutive/randomly selected from case population with clearly defined random frame | 2 |
| 1. Consecutive/randomly selected from case population without clearly defined random frame or with extensive inclusion criteria | 1 |
| 1. Method of selection not described | 0 |
| ***Representativeness of controls*** | |
| 1. Controls were consecutive/randomly drawn from the same area (ward/community) as cases with the same criteria | 2 |
| 1. Controls were consecutive/randomly drawn from a different area than cases | 1 |
| 1. Not described | 0 |
| ***Ascertainment of cancer cases*** | |
| 1. Clearly described objective criteria for diagnosis of cancer | 1 |
| 1. Not described | 0 |
| ***Ascertainment of controls*** | |
| 1. Clinical examinations were performed on controls to prove that controls did not have cancer | 2 |
| 1. Article merely stated that controls were subjects who did not have cancer; no proof provided | 1 |
| 1. Not described | 0 |
| ***Ascertainment of genotyping examination*** | |
| 1. Genotyping done under “blind” conditions | 1 |
| 1. Unblended or not mentioned | 0 |
| ***Test for Hardy-Weinberg equilibrium*** | |
| 1. Hardy-Weinberg equilibrium in control group | 2 |
| 1. Hardy-Weinberg disequilibrium in control group | 1 |
| 1. Hardy-Weinberg equilibrium not checked | 0 |
| ***Association assessment*** | |
| 1. Assessed association between genotypes and cancer with appropriate statistic and adjusting confounders | 2 |
| 1. Assessed association between genotypes and cancer with appropriate statistic without adjusting confounders | 1 |
| 1. Inappropriate statistic used | 0 |
