# Supplementary material for: APEX Nuclease (Multifunctional DNA Repair Enzyme) 1 Gene Asp148Glu Polymorphism and Cancer Risk: A Meta-Analysis Involving 58 Articles and 48903 Participants
Source: PLoS One. 2013 Dec 12;8(12):e83527. doi: 10.1371/journal.pone.0083527 (PMC3861501; doi:10.1371/journal.pone.0083527)
Supplement: Table S2 — The genotype distributions and allele frequencies of the APEX1 Asp148Glu polymorphism between cancer patients and controls of all examined populations in this meta-analysis. (DOC) [file pone.0083527.s002.doc]

**Supplementary Table S2.** The genotype distributions and allele frequencies of the *APEX1* Asp148Glu polymorphism between cancer patients and controls of all examined populations in this meta-analysis

| **Author (publication year)** | **Cases** | | | |  | **Controls** | | | |
| --- | --- | --- | --- | --- | --- | --- | --- | --- | --- |
|  | Asp/Asp | Asp/Glu | Glu/Glu | Glu (%) |  | Asp/Asp | Asp/Glu | Glu/Glu | Glu (%) |
| Misra RR et al (2003) | 64 | 167 | 79 | 52.42 |  | 65 | 160 | 77 | 51.99 |
| Popanda O et al (2004) | 135 | 235 | 89 | 44.99 |  | 118 | 233 | 106 | 48.69 |
| Ito H et al (2004) | 62 | 84 | 32 | 41.57 |  | 159 | 226 | 64 | 39.42 |
| Chen L et al (2005) | 65 | 122 | 41 | 44.74 |  | 73 | 108 | 36 | 41.47 |
| Chen L et al (2005) | 42 | 64 | 17 | 39.84 |  | 42 | 59 | 11 | 36.16 |
| Shen M et al (2005) | 30 | 61 | 26 | 48.29 |  | 37 | 61 | 15 | 40.27 |
| Broberg K et al (2005) | 9 | 35 | 17 | 56.56 |  | 37 | 79 | 39 | 50.65 |
| Li C et al (2006) | 188 | 273 | 141 | 46.10 |  | 156 | 297 | 150 | 49.50 |
| Zienolddiny S et al (2006) | 117 | 67 | 80 | 42.99 |  | 138 | 60 | 122 | 47.50 |
| Moreno V et al (2006) | 95 | 177 | 87 | 48.89 |  | 99 | 147 | 66 | 44.71 |
| Zhang Y et al (2006) (Postmenopausal) | 218 | 414 | 207 | 49.34 |  | 187 | 330 | 162 | 48.16 |
| Zhang Y et al (2006) (Premenopausal) | 161 | 284 | 142 | 48.38 |  | 116 | 210 | 108 | 49.08 |
| Terry PD et al (2006) | 51 | 133 | 45 | 48.69 |  | 63 | 104 | 40 | 44.44 |
| Li J et al (2006) | 108 | 180 | 79 | 46.05 |  | 85 | 174 | 71 | 47.88 |
| Huang M et al (2007) | 176 | 420 | 0 | 35.23 |  | 166 | 424 | 0 | 35.93 |
| Berndt S et al (2007) | 175 | 364 | 153 | 48.41 |  | 204 | 335 | 171 | 47.68 |
| Li C et al (2007) | 217 | 428 | 185 | 48.07 |  | 234 | 437 | 183 | 47.01 |
| Berndt S et al (2007) | 186 | 387 | 166 | 48.65 |  | 222 | 357 | 178 | 47.09 |
| Figueroa JD et al (2007) | 335 | 510 | 249 | 46.07 |  | 292 | 491 | 230 | 46.94 |
| De Ruyck K et al (2007) | 21 | 60 | 29 | 53.64 |  | 41 | 41 | 28 | 44.09 |
| Smith TR et al (2008) | 103 | 140 | 76 | 45.77 |  | 104 | 209 | 92 | 48.52 |
| Andrew AS et al (2008) | 259 | 461 | 191 | 46.27 |  | 333 | 586 | 246 | 46.27 |
| Kasahara M et al (2008) | 23 | 45 | 0 | 33.09 |  | 70 | 51 | 0 | 21.07 |
| Shekari M et al (2008) | 94 | 40 | 4 | 17.39 |  | 94 | 73 | 13 | 27.50 |
| Sangrajrang S et al (2008) (Premenopausal) | 135 | 110 | 23 | 29.10 |  | 114 | 105 | 26 | 32.04 |
| Zhu R et al (2008) | 31 | 48 | 26 | 47.62 |  | 48 | 49 | 11 | 32.87 |
| Huang WY et al (2008) | 136 | 202 | 71 | 42.05 |  | 237 | 376 | 170 | 45.72 |
| Pardini B et al (2008) | 140 | 261 | 130 | 49.06 |  | 157 | 267 | 106 | 45.19 |
| Chiang FY et al (2008) | 102 | 130 | 51 | 40.99 |  | 179 | 214 | 76 | 39.02 |
| Mitra AK et al (2008) | 62 | 75 | 13 | 33.67 |  | 144 | 72 | 9 | 20.00 |
| Sangrajrang S et al (2008) (Postmenopausal) | 115 | 98 | 26 | 31.38 |  | 80 | 71 | 29 | 35.83 |
| Smith TR et al (2008) | 23 | 22 | 8 | 35.85 |  | 30 | 33 | 12 | 38.00 |
| Chang JS et al (2008) | 111 | 104 | 40 | 36.08 |  | 110 | 129 | 41 | 37.68 |
| Chang JS et al (2008) | 38 | 61 | 14 | 39.38 |  | 102 | 141 | 56 | 42.31 |
| Tse D et al (2008) | 75 | 162 | 74 | 49.84 |  | 123 | 228 | 103 | 47.80 |
| Liu Y et al (2009) | 81 | 289 | 0 | 39.05 |  | 101 | 262 | 0 | 36.09 |
| Lu J et al (2009) | 182 | 228 | 90 | 40.80 |  | 176 | 265 | 76 | 40.33 |
| Agachan B et al (2009) | 38 | 40 | 20 | 40.82 |  | 45 | 17 | 5 | 20.15 |
| Ji L et al (2009) | 455 | 37 | 15 | 6.61 |  | 402 | 35 | 63 | 16.10 |
| Gangwar R et al (2009) | 122 | 80 | 4 | 21.36 |  | 141 | 92 | 17 | 25.20 |
| Lo YL et al (2009) | 261 | 349 | 119 | 40.26 |  | 272 | 332 | 118 | 39.34 |
| Wang MM et al (2010) | 94 | 146 | 66 | 45.42 |  | 89 | 145 | 72 | 47.22 |
| Jelonek K et al (2010) | 16 | 50 | 25 | 54.95 |  | 90 | 223 | 99 | 51.09 |
| Palli D et al (2010) | 103 | 147 | 48 | 40.77 |  | 208 | 243 | 95 | 39.65 |
| Jelonek K et al (2010) | 49 | 59 | 5 | 30.53 |  | 38 | 87 | 28 | 46.73 |
| Brevik A et al (2010) | 102 | 137 | 65 | 43.91 |  | 108 | 167 | 84 | 46.66 |
| Agalliu I et al (2010) | 57 | 65 | 20 | 36.97 |  | 37 | 33 | 9 | 32.28 |
| Jelonek K et al (2010) | 28 | 56 | 20 | 46.15 |  | 22 | 66 | 22 | 50.00 |
| Canbay E et al (2010) | 14 | 18 | 8 | 42.50 |  | 151 | 63 | 33 | 26.11 |
| Osawa K et al (2010) | 41 | 63 | 0 | 30.29 |  | 72 | 48 | 0 | 20.00 |
| Agalliu I et al (2010) | 424 | 612 | 229 | 42.29 |  | 415 | 615 | 212 | 41.83 |
| Wang M et al (2010) | 78 | 116 | 40 | 41.88 |  | 84 | 129 | 40 | 41.30 |
| Ye CC et al (2010) | 37 | 86 | 0 | 34.96 |  | 52 | 106 | 0 | 33.54 |
| Kuasne H et al (2011) | 84 | 83 | 5 | 27.03 |  | 106 | 64 | 2 | 19.77 |
| Gu D et al (2011) | 69 | 185 | 84 | 52.22 |  | 110 | 183 | 69 | 44.34 |
| Zhonghua L et al (2011) | 26 | 64 | 37 | 54.33 |  | 56 | 70 | 30 | 41.67 |
| Cao Q et al (2011) | 181 | 292 | 139 | 46.57 |  | 199 | 329 | 104 | 42.48 |
| Huang LZ et al (2011) | 141 | 191 | 83 | 43.01 |  | 170 | 253 | 96 | 42.87 |
| Li Z et al (2011) | 179 | 199 | 77 | 38.79 |  | 172 | 213 | 58 | 37.13 |
| Deng Q et al (2011) | 123 | 143 | 49 | 38.25 |  | 97 | 159 | 58 | 43.79 |
| Canbay E et al (2011) | 28 | 43 | 8 | 37.34 |  | 151 | 63 | 33 | 26.11 |
| Cao Q et al (2011) | 303 | 87 | 0 | 11.15 |  | 350 | 59 | 0 | 7.21 |
| Mandal R et al (2012) | 106 | 71 | 15 | 26.30 |  | 118 | 94 | 12 | 26.34 |
| Cincin Z et al (2012) | 40 | 9 | 55 | 57.21 |  | 87 | 16 | 55 | 39.87 |
| Mittal RD et al (2012) | 108 | 72 | 15 | 26.15 |  | 136 | 101 | 13 | 25.40 |
| Mittal RD et al (2012) | 126 | 82 | 4 | 21.23 |  | 141 | 92 | 17 | 25.20 |
| Nakao M et al (2012) | 77 | 75 | 33 | 38.11 |  | 542 | 681 | 242 | 39.76 |
| Li Y et al (2013) | 123 | 247 | 81 | 45.34 |  | 186 | 335 | 110 | 43.98 |
