# Supplementary material for: APEX Nuclease (Multifunctional DNA Repair Enzyme) 1 Gene Asp148Glu Polymorphism and Cancer Risk: A Meta-Analysis Involving 58 Articles and 48903 Participants
Source: PLoS One. 2013 Dec 12;8(12):e83527. doi: 10.1371/journal.pone.0083527 (PMC3861501; doi:10.1371/journal.pone.0083527)
Supplement: Figure S1 — Forest plots of the lung, bladder, colorectal cancer (the upper panel), and prostate, breast, gastric cancers (the lower panel) in subgroup analyses by cancer type for the APEX1 Asp148Glu polymorphism under the allelic model. (PDF) [file pone.0083527.s003.pdf]

**Supplementary Figure S1.** Forest plots of the lung, bladder, colorectal cancer (the upper panel), and prostate, breast, gastric cancers (the lower panel) in subgroup analyses by cancer type for the *APEX1* Asp148Glu polymorphism under the allelic model

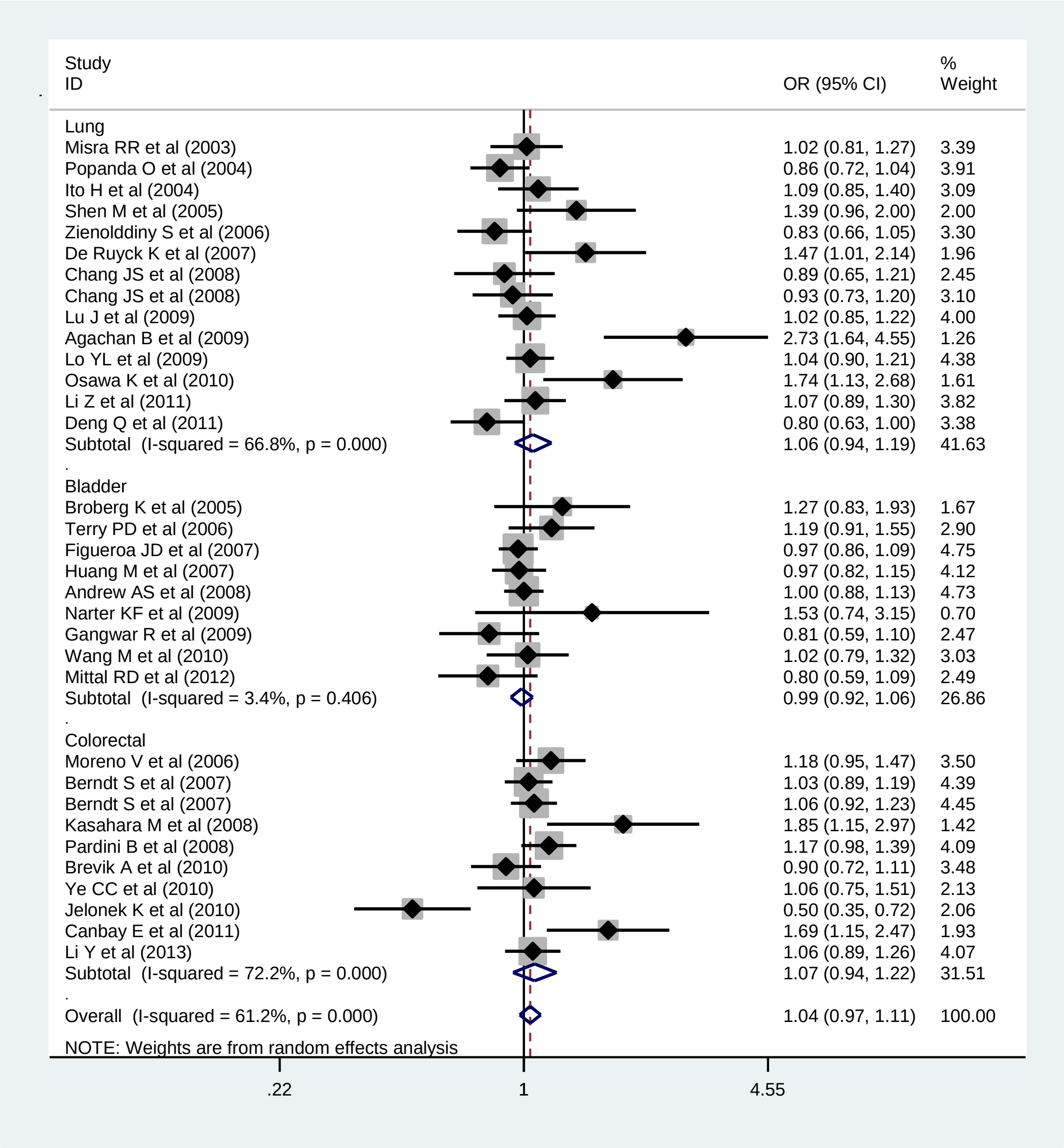

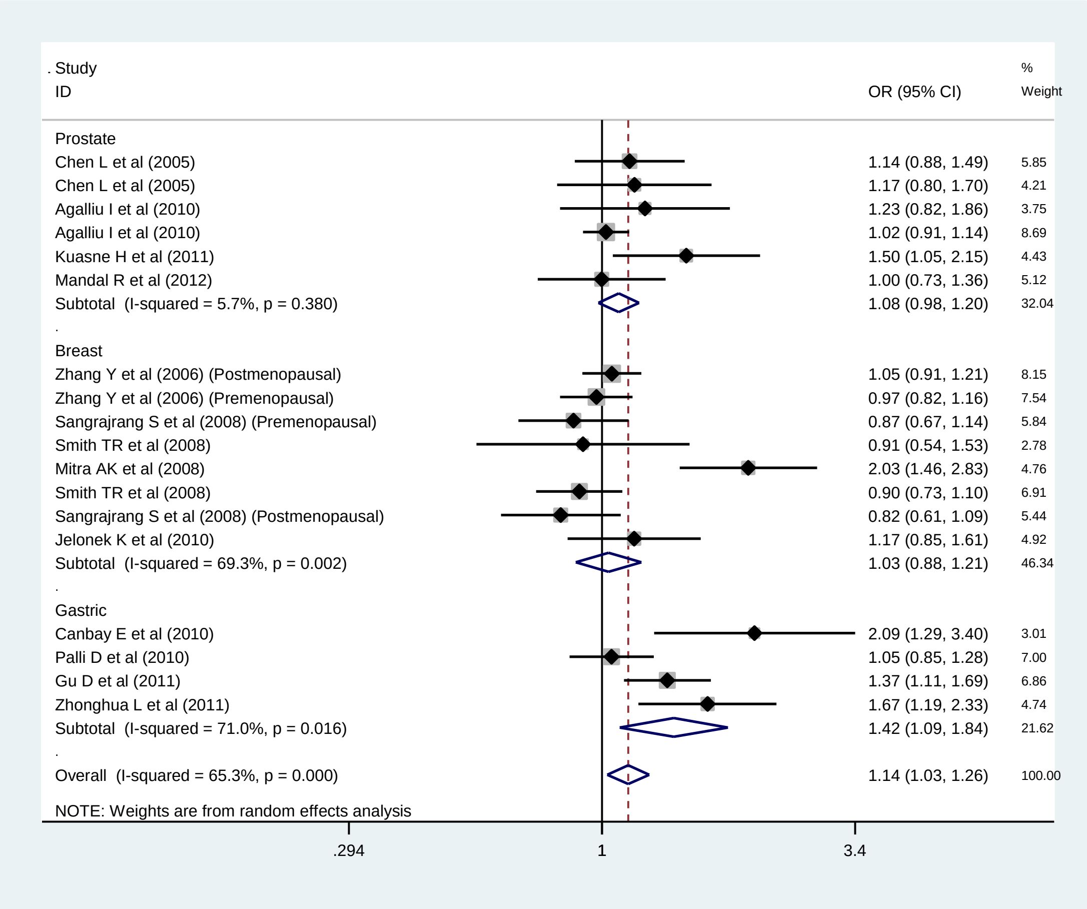

. Study
ID
OR (95% CI)
% Weight
Prostate
Chen L et al (2005) 1.14 (0.88, 1.49) 5.85
Chen L et al (2005) 1.17 (0.80, 1.70) 4.21
Agalliu I et al (2010) 1.23 (0.82, 1.86) 3.75
Agalliu I et al (2010) 1.02 (0.91, 1.14) 8.69
Kuasne H et al (2011) 1.50 (1.05, 2.15) 4.43
Mandal R et al (2012) 1.00 (0.73, 1.36) 5.12
Subtotal (I-squared = 5.7%, p = 0.380) 1.08 (0.98, 1.20) 32.04
Breast
Zhang Y et al (2006) (Postmenopausal) 1.05 (0.91, 1.21) 8.15
Zhang Y et al (2006) (Premenopausal) 0.97 (0.82, 1.16) 7.54
Sangrajrang S et al (2008) (Premenopausal) 0.87 (0.67, 1.14) 5.84
Smith TR et al (2008) 0.91 (0.54, 1.53) 2.78
Mitra AK et al (2008) 2.03 (1.46, 2.83) 4.76
Smith TR et al (2008) 0.90 (0.73, 1.10) 6.91
Sangrajrang S et al (2008) (Postmenopausal) 0.82 (0.61, 1.09) 5.44
Jelonek K et al (2010) 1.17 (0.85, 1.61) 4.92
Subtotal (I-squared = 69.3%, p = 0.002) 1.03 (0.88, 1.21) 46.34
Gastric
Canbay E et al (2010) 2.09 (1.29, 3.40) 3.01
Palli D et al (2010) 1.05 (0.85, 1.28) 7.00
Gu D et al (2011) 1.37 (1.11, 1.69) 6.86
Zhonghua L et al (2011) 1.67 (1.19, 2.33) 4.74
Subtotal (I-squared = 71.0%, p = 0.016) 1.42 (1.09, 1.84) 21.62
Overall (I-squared = 65.3%, p = 0.000) 1.14 (1.03, 1.26) 100.00
NOTE: Weights are from random effects analysis
.294
1
3.4
