# Supplementary material for: APEX Nuclease (Multifunctional DNA Repair Enzyme) 1 Gene Asp148Glu Polymorphism and Cancer Risk: A Meta-Analysis Involving 58 Articles and 48903 Participants
Source: PLoS One. 2013 Dec 12;8(12):e83527. doi: 10.1371/journal.pone.0083527 (PMC3861501; doi:10.1371/journal.pone.0083527)
Supplement: Figure S2 — Forest plots of the Caucasians (the upper panel), Asians (the middle panel), African-Americans and mixed populations (the lower panel) in subgroup analyses by ethnicity for the APEX1 Asp148Glu polymorphism under the allelic model. (PDF) [file pone.0083527.s004.pdf]

Supplementary Figure S2. Forest plots of the Caucasians (the upper panel), Asians (the middle panel), African-Americans and mixed populations (the lower panel) in subgroup analyses by ethnicity for the *APEX1* Asp148Glu polymorphism under the allelic model

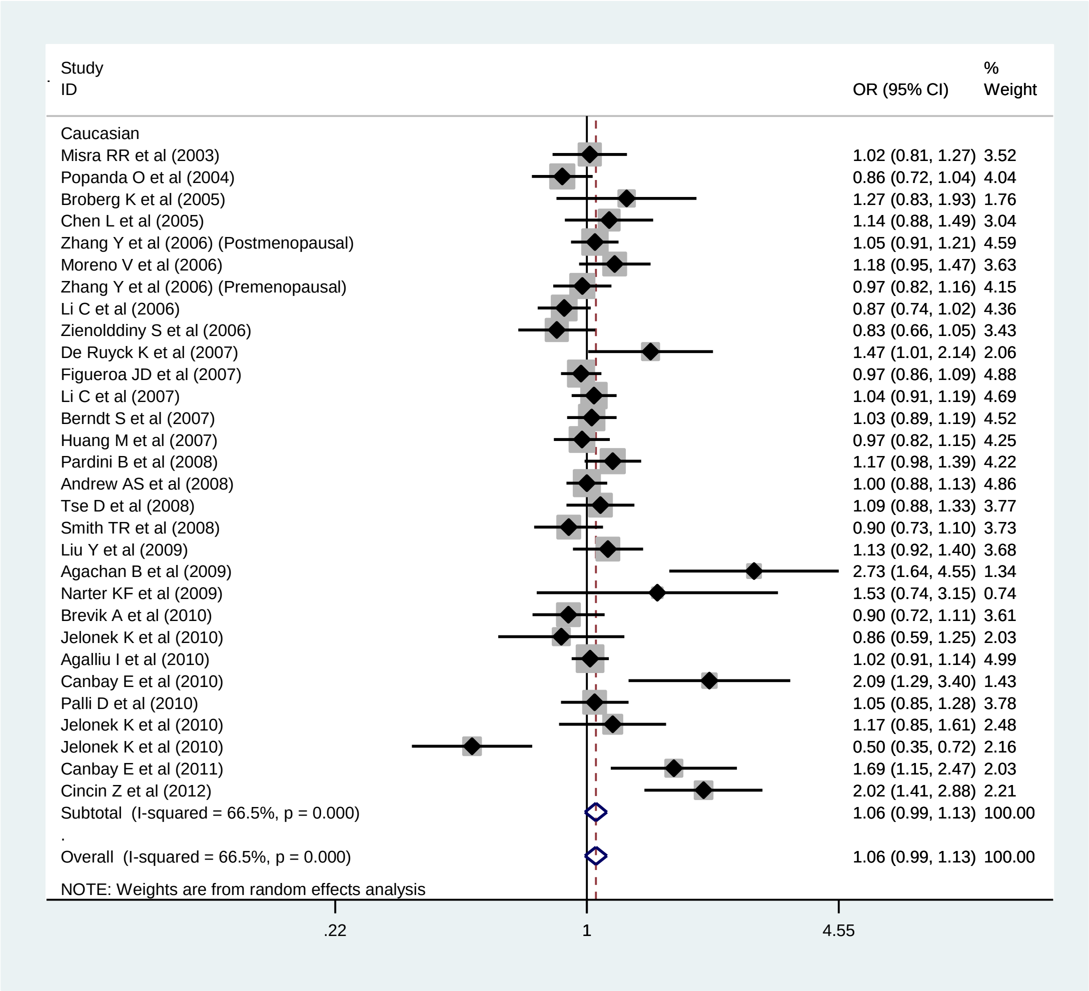

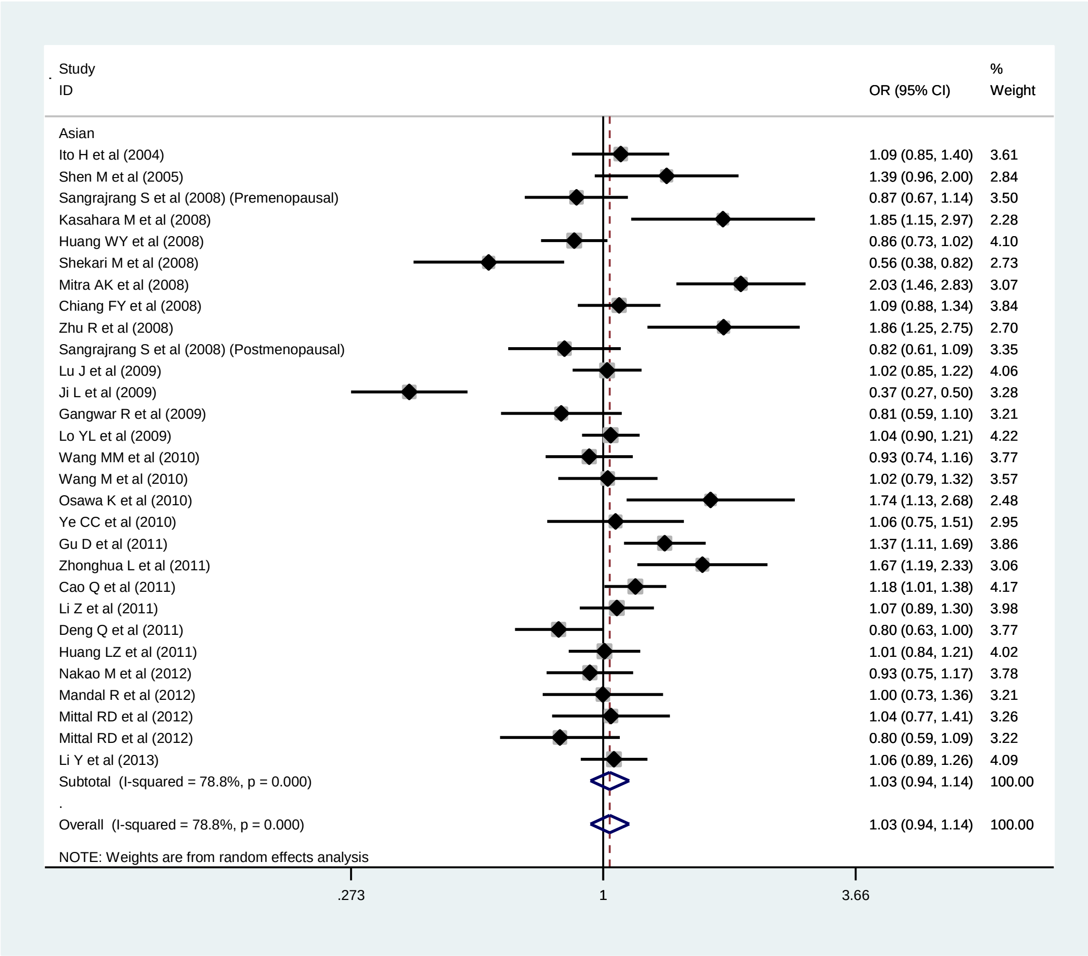
Study
ID
OR (95% CI)
%
Weight
Asian
Ito H et al (2004) 1.09 (0.85, 1.40) 3.61
Shen M et al (2005) 1.39 (0.96, 2.00) 2.84
Sangrajrang S et al (2008) (Premenopausal) 0.87 (0.67, 1.14) 3.50
Kasahara M et al (2008) 1.85 (1.15, 2.97) 2.28
Huang WY et al (2008) 0.86 (0.73, 1.02) 4.10
Shekari M et al (2008) 0.56 (0.38, 0.82) 2.73
Mitra AK et al (2008) 2.03 (1.46, 2.83) 3.07
Chiang FY et al (2008) 1.09 (0.88, 1.34) 3.84
Zhu R et al (2008) 1.86 (1.25, 2.75) 2.70
Sangrajrang S et al (2008) (Postmenopausal) 0.82 (0.61, 1.09) 3.35
Lu J et al (2009) 1.02 (0.85, 1.22) 4.06
Ji L et al (2009) 0.37 (0.27, 0.50) 3.28
Gangwar R et al (2009) 0.81 (0.59, 1.10) 3.21
Lo YL et al (2009) 1.04 (0.90, 1.21) 4.22
Wang MM et al (2010) 0.93 (0.74, 1.16) 3.77
Wang M et al (2010) 1.02 (0.79, 1.32) 3.57
Osawa K et al (2010) 1.74 (1.13, 2.68) 2.48
Ye CC et al (2010) 1.06 (0.75, 1.51) 2.95
Gu D et al (2011) 1.37 (1.11, 1.69) 3.86
Zhonghua L et al (2011) 1.67 (1.19, 2.33) 3.06
Cao Q et al (2011) 1.18 (1.01, 1.38) 4.17
Li Z et al (2011) 1.07 (0.89, 1.30) 3.98
Deng Q et al (2011) 0.80 (0.63, 1.00) 3.77
Huang LZ et al (2011) 1.01 (0.84, 1.21) 4.02
Nakao M et al (2012) 0.93 (0.75, 1.17) 3.78
Mandal R et al (2012) 1.00 (0.73, 1.36) 3.21
Mittal RD et al (2012) 1.04 (0.77, 1.41) 3.26
Mittal RD et al (2012) 0.80 (0.59, 1.09) 3.22
Li Y et al (2013) 1.06 (0.89, 1.26) 4.09
Subtotal (I-squared = 78.8%, p = 0.000) 1.03 (0.94, 1.14) 100.00
Overall (I-squared = 78.8%, p = 0.000) 1.03 (0.94, 1.14) 100.00
NOTE: Weights are from random effects analysis
.273
1
3.66

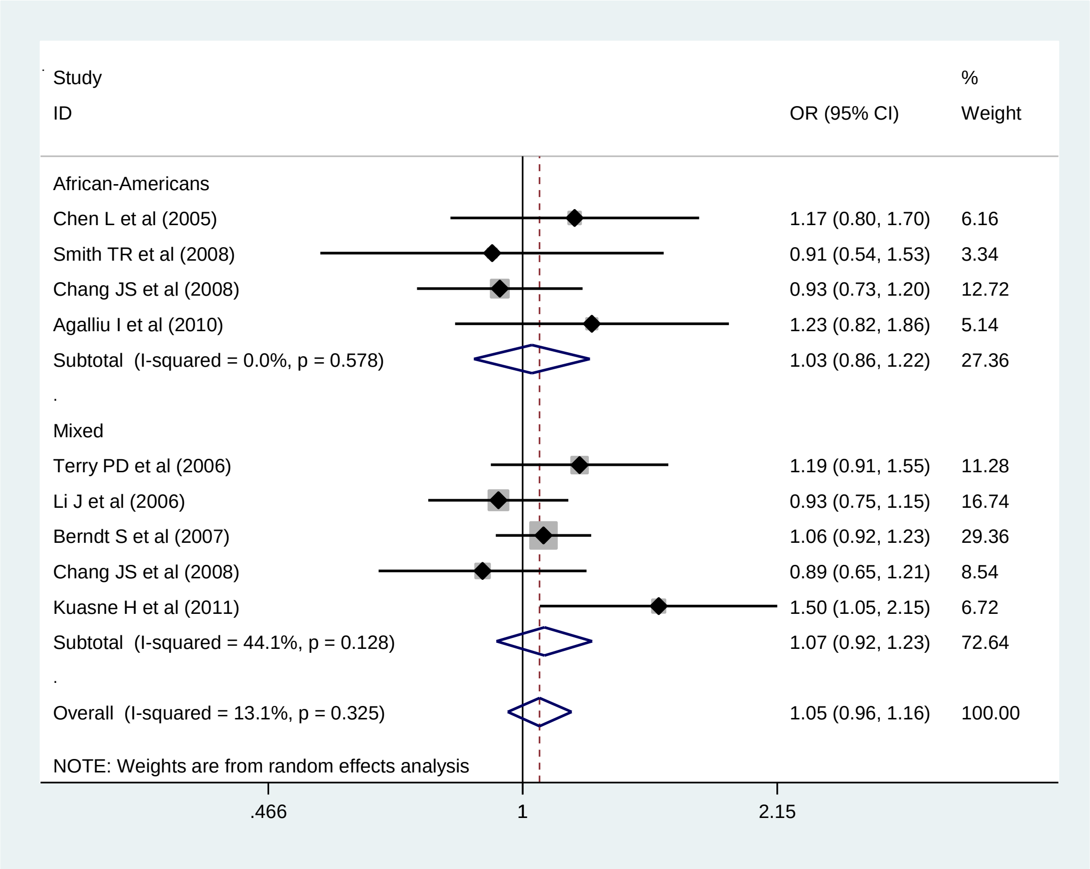
Study
ID
OR (95% CI)
%
Weight
African-Americans
Chen L et al (2005)
1.17 (0.80, 1.70)
6.16
Smith TR et al (2008)
0.91 (0.54, 1.53)
3.34
Chang JS et al (2008)
0.93 (0.73, 1.20)
12.72
Agalliu I et al (2010)
1.23 (0.82, 1.86)
5.14
Subtotal (I-squared = 0.0%, p = 0.578)
1.03 (0.86, 1.22)
27.36
Mixed
Terry PD et al (2006)
1.19 (0.91, 1.55)
11.28
Li J et al (2006)
0.93 (0.75, 1.15)
16.74
Berndt S et al (2007)
1.06 (0.92, 1.23)
29.36
Chang JS et al (2008)
0.89 (0.65, 1.21)
8.54
Kuasne H et al (2011)
1.50 (1.05, 2.15)
6.72
Subtotal (I-squared = 44.1%, p = 0.128)
1.07 (0.92, 1.23)
72.64
Overall (I-squared = 13.1%, p = 0.325)
1.05 (0.96, 1.16)
100.00
NOTE: Weights are from random effects analysis
.466
1
2.15
