# Supplementary material for: APEX Nuclease (Multifunctional DNA Repair Enzyme) 1 Gene Asp148Glu Polymorphism and Cancer Risk: A Meta-Analysis Involving 58 Articles and 48903 Participants
Source: PLoS One. 2013 Dec 12;8(12):e83527. doi: 10.1371/journal.pone.0083527 (PMC3861501; doi:10.1371/journal.pone.0083527)
Supplement: Figure S3 — Forest plots of the hospital-based studies (the upper panel), and population-based studies (the lower panel) in subgroup analyses by study design for the APEX1 Asp148Glu polymorphism under the allelic model. (PDF) [file pone.0083527.s005.pdf]

Supplementary Figure S3. Forest plots of the hospital-based studies (the upper panel), and population-based studies (the lower panel) in subgroup analyses by study design for the *APEX1* Asp148Glu polymorphism under the allelic model

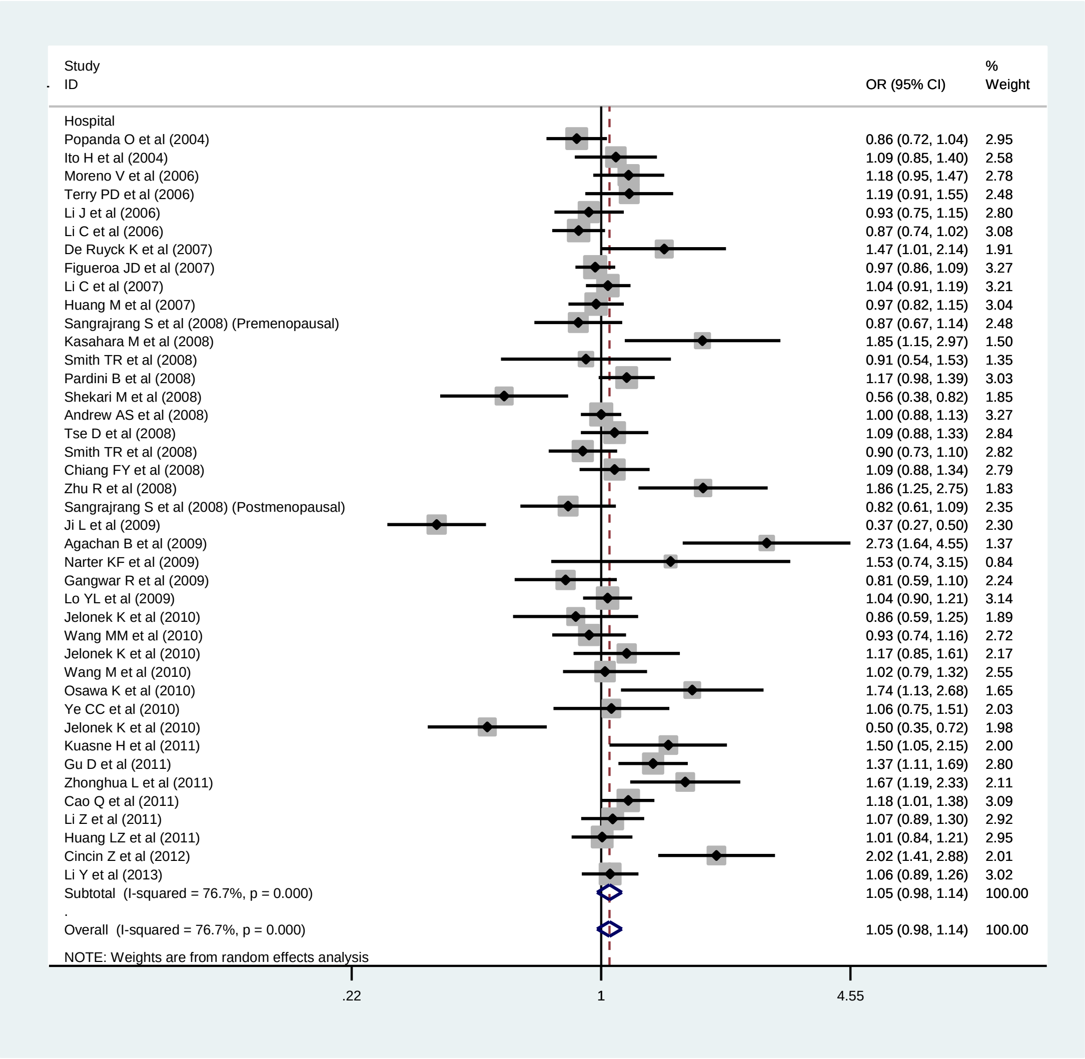

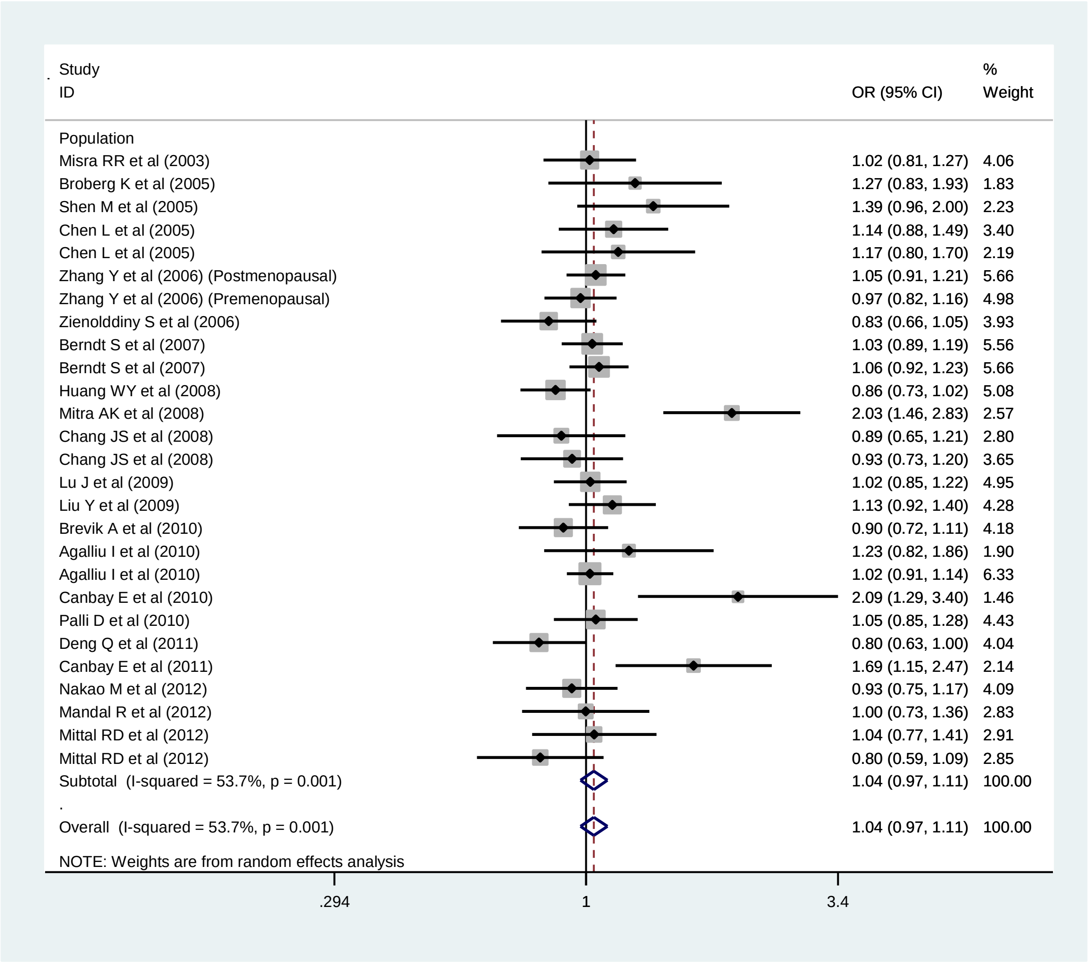

Study
ID
OR (95% CI)
%
Weight
Population
Misra RR et al (2003) 1.02 (0.81, 1.27) 4.06
Broberg K et al (2005) 1.27 (0.83, 1.93) 1.83
Shen M et al (2005) 1.39 (0.96, 2.00) 2.23
Chen L et al (2005) 1.14 (0.88, 1.49) 3.40
Chen L et al (2005) 1.17 (0.80, 1.70) 2.19
Zhang Y et al (2006) (Postmenopausal) 1.05 (0.91, 1.21) 5.66
Zhang Y et al (2006) (Premenopausal) 0.97 (0.82, 1.16) 4.98
Zienolddiny S et al (2006) 0.83 (0.66, 1.05) 3.93
Berndt S et al (2007) 1.03 (0.89, 1.19) 5.56
Berndt S et al (2007) 1.06 (0.92, 1.23) 5.66
Huang WY et al (2008) 0.86 (0.73, 1.02) 5.08
Mitra AK et al (2008) 2.03 (1.46, 2.83) 2.57
Chang JS et al (2008) 0.89 (0.65, 1.21) 2.80
Chang JS et al (2008) 0.93 (0.73, 1.20) 3.65
Lu J et al (2009) 1.02 (0.85, 1.22) 4.95
Liu Y et al (2009) 1.13 (0.92, 1.40) 4.28
Brevik A et al (2010) 0.90 (0.72, 1.11) 4.18
Agalliu I et al (2010) 1.23 (0.82, 1.86) 1.90
Agalliu I et al (2010) 1.02 (0.91, 1.14) 6.33
Canbay E et al (2010) 2.09 (1.29, 3.40) 1.46
Palli D et al (2010) 1.05 (0.85, 1.28) 4.43
Deng Q et al (2011) 0.80 (0.63, 1.00) 4.04
Canbay E et al (2011) 1.69 (1.15, 2.47) 2.14
Nakao M et al (2012) 0.93 (0.75, 1.17) 4.09
Mandal R et al (2012) 1.00 (0.73, 1.36) 2.83
Mittal RD et al (2012) 1.04 (0.77, 1.41) 2.91
Mittal RD et al (2012) 0.80 (0.59, 1.09) 2.85
Subtotal (I-squared = 53.7%, p = 0.001) 1.04 (0.97, 1.11) 100.00
.
Overall (I-squared = 53.7%, p = 0.001) 1.04 (0.97, 1.11) 100.00
NOTE: Weights are from random effects analysis
.294
1
3.4
