# Supplementary material for: APEX Nuclease (Multifunctional DNA Repair Enzyme) 1 Gene Asp148Glu Polymorphism and Cancer Risk: A Meta-Analysis Involving 58 Articles and 48903 Participants
Source: PLoS One. 2013 Dec 12;8(12):e83527. doi: 10.1371/journal.pone.0083527 (PMC3861501; doi:10.1371/journal.pone.0083527)
Supplement: Figure S4 — Forest plots of the small studies (the upper panel), and large studies (the lower panel) in subgroup analyses by sample size for the APEX1 Asp148Glu polymorphism under the allelic model. (PDF) [file pone.0083527.s006.pdf]

Supplementary Figure S4. Forest plots of the small studies (the upper panel), and large studies (the lower panel) in subgroup analyses by sample size for the *APEX1* Asp148Glu polymorphism under the allelic model

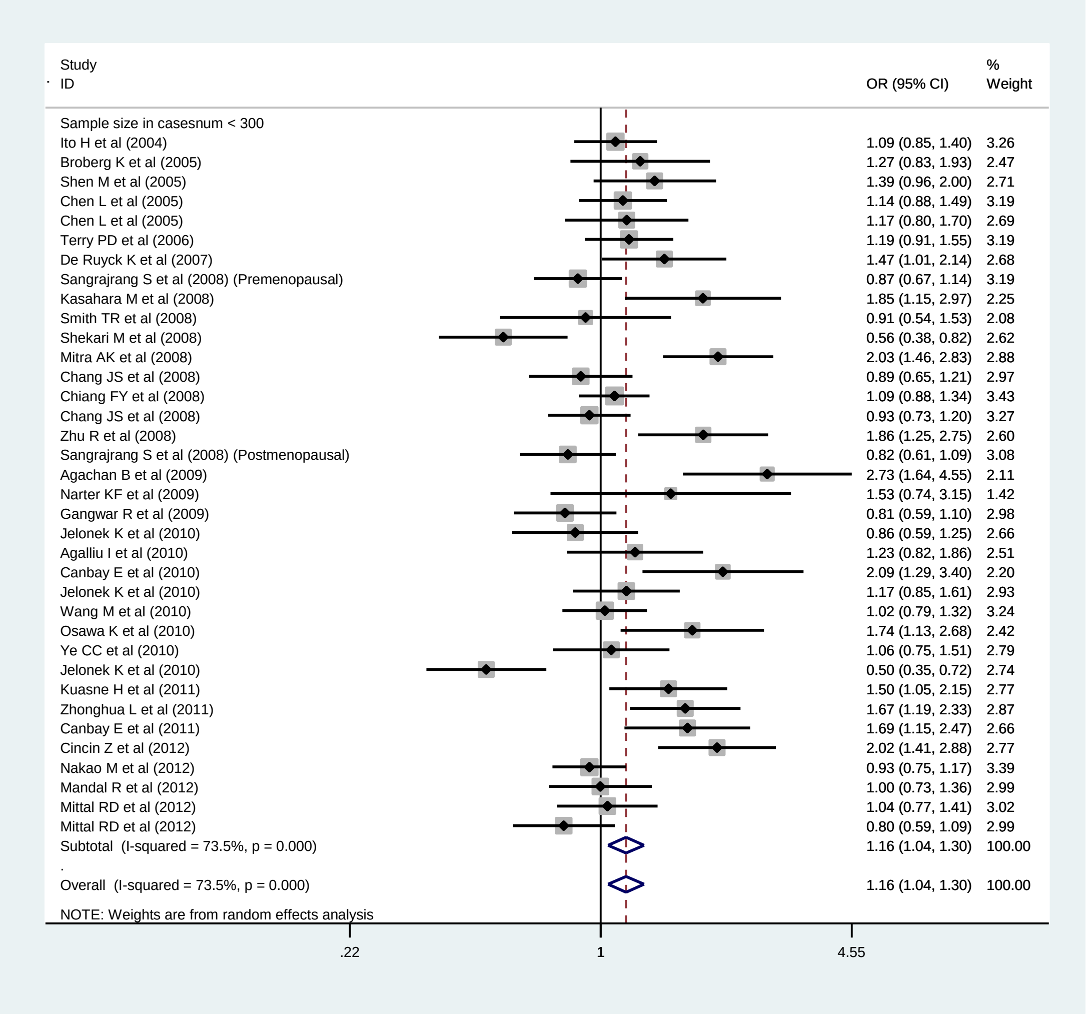

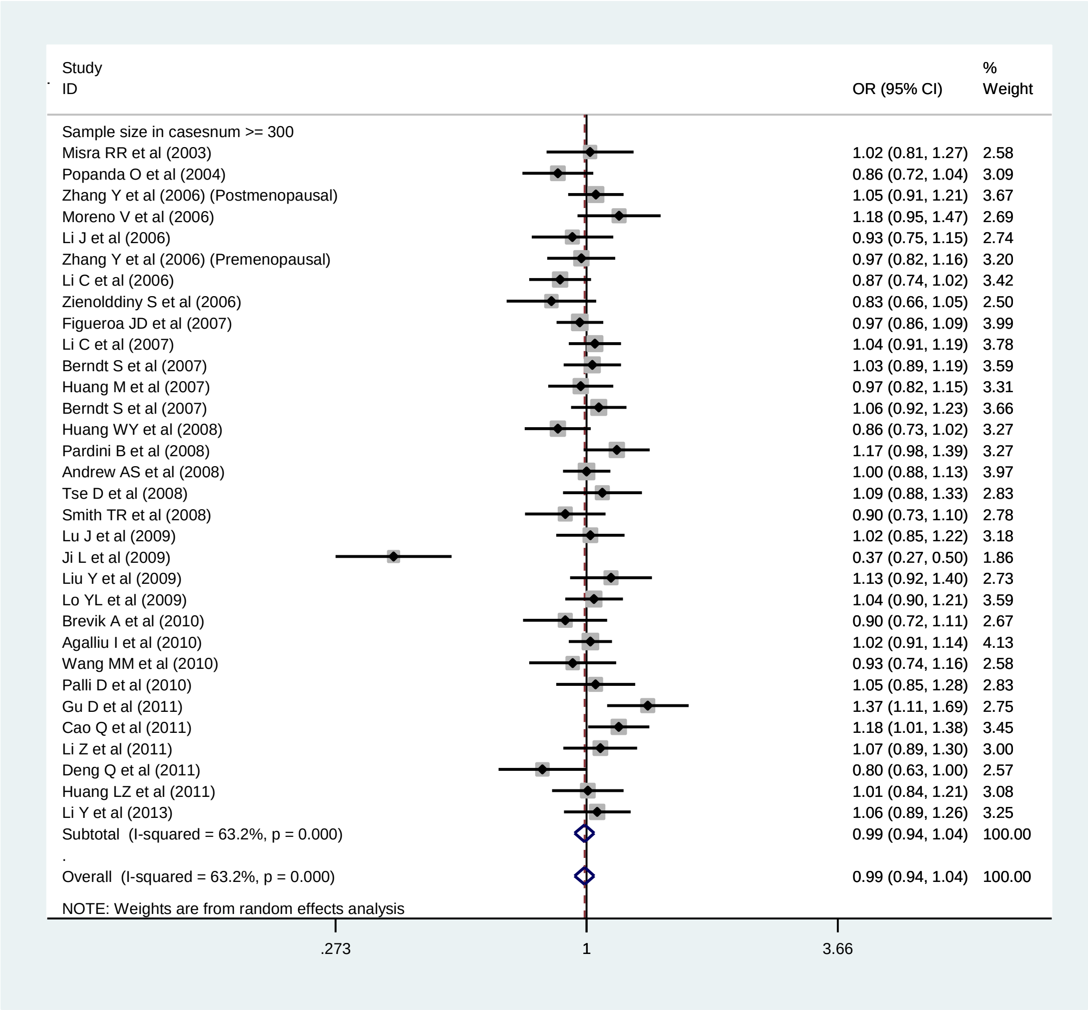
Study
ID
OR (95% CI)
%
Weight
Sample size in casesnum >= 300
Misra RR et al (2003) 1.02 (0.81, 1.27) 2.58
Popanda O et al (2004) 0.86 (0.72, 1.04) 3.09
Zhang Y et al (2006) (Postmenopausal) 1.05 (0.91, 1.21) 3.67
Moreno V et al (2006) 1.18 (0.95, 1.47) 2.69
Li J et al (2006) 0.93 (0.75, 1.15) 2.74
Zhang Y et al (2006) (Premenopausal) 0.97 (0.82, 1.16) 3.20
Li C et al (2006) 0.87 (0.74, 1.02) 3.42
Zienolddiny S et al (2006) 0.83 (0.66, 1.05) 2.50
Figueroa JD et al (2007) 0.97 (0.86, 1.09) 3.99
Li C et al (2007) 1.04 (0.91, 1.19) 3.78
Berndt S et al (2007) 1.03 (0.89, 1.19) 3.59
Huang M et al (2007) 0.97 (0.82, 1.15) 3.31
Berndt S et al (2007) 1.06 (0.92, 1.23) 3.66
Huang WY et al (2008) 0.86 (0.73, 1.02) 3.27
Pardini B et al (2008) 1.17 (0.98, 1.39) 3.27
Andrew AS et al (2008) 1.00 (0.88, 1.13) 3.97
Tse D et al (2008) 1.09 (0.88, 1.33) 2.83
Smith TR et al (2008) 0.90 (0.73, 1.10) 2.78
Lu J et al (2009) 1.02 (0.85, 1.22) 3.18
Ji L et al (2009) 0.37 (0.27, 0.50) 1.86
Liu Y et al (2009) 1.13 (0.92, 1.40) 2.73
Lo YL et al (2009) 1.04 (0.90, 1.21) 3.59
Brevik A et al (2010) 0.90 (0.72, 1.11) 2.67
Agalliu I et al (2010) 1.02 (0.91, 1.14) 4.13
Wang MM et al (2010) 0.93 (0.74, 1.16) 2.58
Palli D et al (2010) 1.05 (0.85, 1.28) 2.83
Gu D et al (2011) 1.37 (1.11, 1.69) 2.75
Cao Q et al (2011) 1.18 (1.01, 1.38) 3.45
Li Z et al (2011) 1.07 (0.89, 1.30) 3.00
Deng Q et al (2011) 0.80 (0.63, 1.00) 2.57
Huang LZ et al (2011) 1.01 (0.84, 1.21) 3.08
Li Y et al (2013) 1.06 (0.89, 1.26) 3.25
Subtotal (I-squared = 63.2%, p = 0.000) 0.99 (0.94, 1.04) 100.00
.
Overall (I-squared = 63.2%, p = 0.000) 0.99 (0.94, 1.04) 100.00
NOTE: Weights are from random effects analysis
.273
1
3.66
