# Supplementary material for: APEX Nuclease (Multifunctional DNA Repair Enzyme) 1 Gene Asp148Glu Polymorphism and Cancer Risk: A Meta-Analysis Involving 58 Articles and 48903 Participants
Source: PLoS One. 2013 Dec 12;8(12):e83527. doi: 10.1371/journal.pone.0083527 (PMC3861501; doi:10.1371/journal.pone.0083527)
Supplement: Figure S5 — Forest plots of the high-quality studies (the upper panel), and low-quality studies (the lower panel) in subgroup analyses by sample size for the APEX1 Asp148Glu polymorphism under the allelic model. (PDF) [file pone.0083527.s007.pdf]

Supplementary Figure S5. Forest plots of the high-quality studies (the upper panel), and low-quality studies (the lower panel) in subgroup analyses by sample size for the *APEX1* Asp148Glu polymorphism under the allelic model

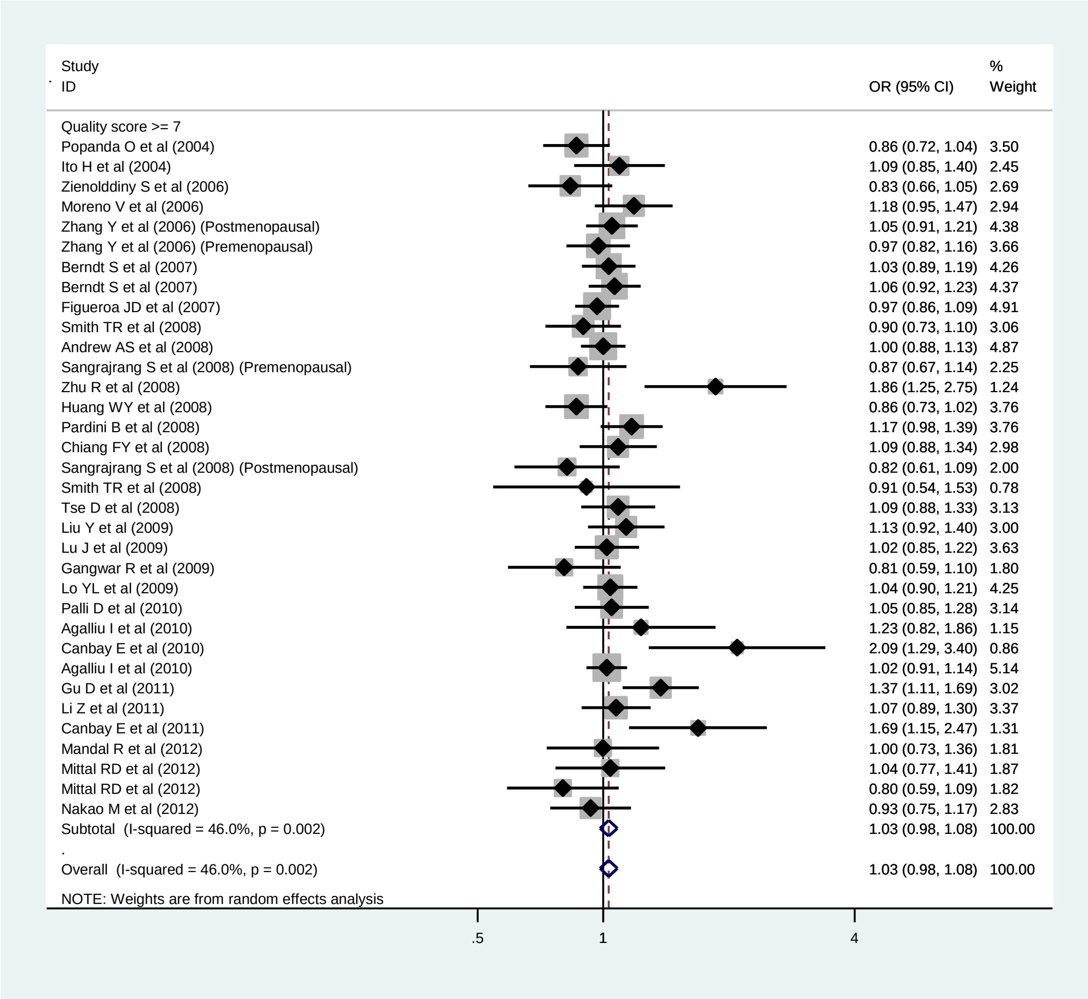

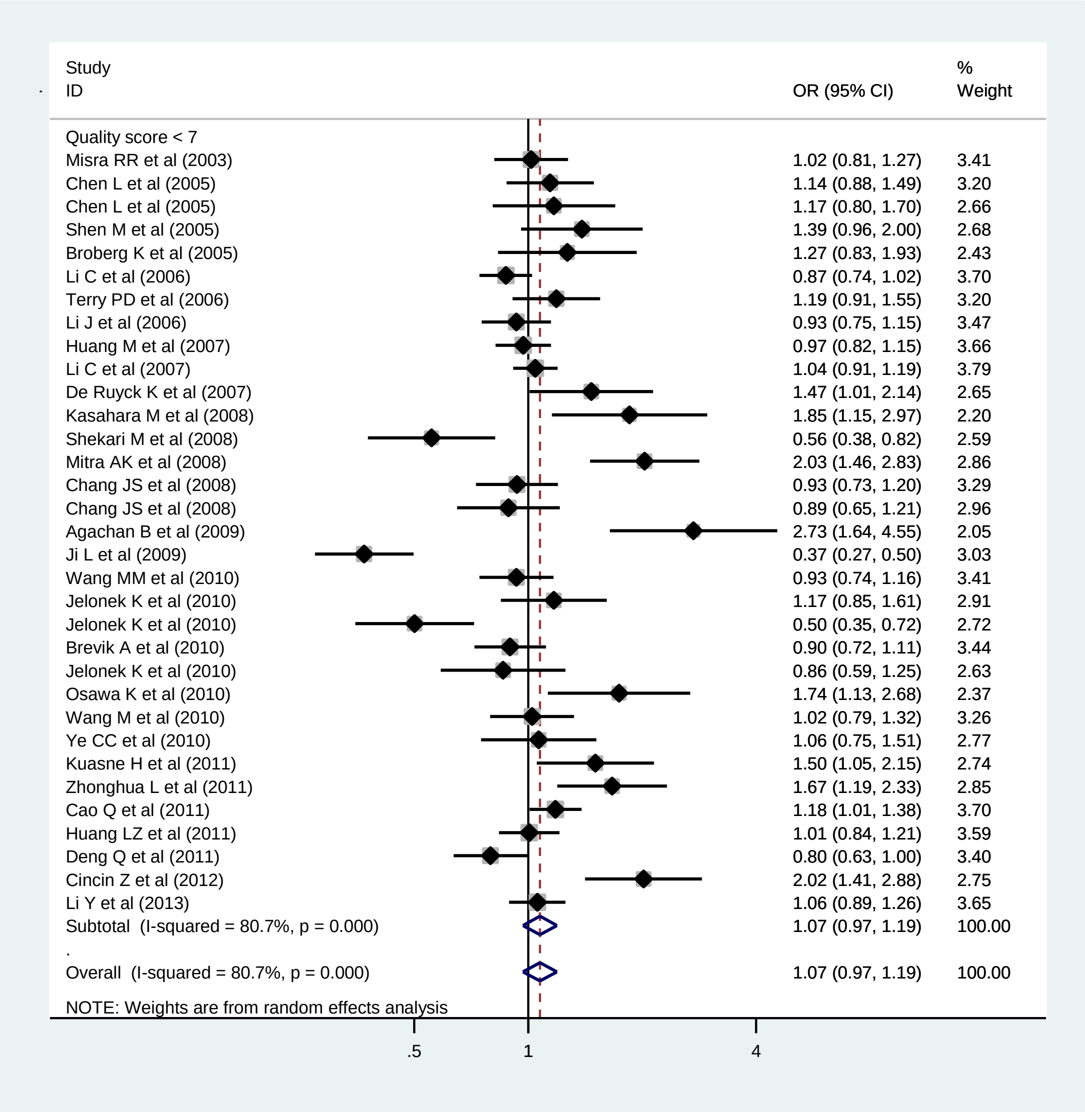
Study
ID
OR (95% CI)
%
Weight
Quality score < 7
Misra RR et al (2003) 1.02 (0.81, 1.27) 3.41
Chen L et al (2005) 1.14 (0.88, 1.49) 3.20
Chen L et al (2005) 1.17 (0.80, 1.70) 2.66
Shen M et al (2005) 1.39 (0.96, 2.00) 2.68
Broberg K et al (2005) 1.27 (0.83, 1.93) 2.43
Li C et al (2006) 0.87 (0.74, 1.02) 3.70
Terry PD et al (2006) 1.19 (0.91, 1.55) 3.20
Li J et al (2006) 0.93 (0.75, 1.15) 3.47
Huang M et al (2007) 0.97 (0.82, 1.15) 3.66
Li C et al (2007) 1.04 (0.91, 1.19) 3.79
De Ruyck K et al (2007) 1.47 (1.01, 2.14) 2.65
Kasahara M et al (2008) 1.85 (1.15, 2.97) 2.20
Shekari M et al (2008) 0.56 (0.38, 0.82) 2.59
Mitra AK et al (2008) 2.03 (1.46, 2.83) 2.86
Chang JS et al (2008) 0.93 (0.73, 1.20) 3.29
Chang JS et al (2008) 0.89 (0.65, 1.21) 2.96
Agachan B et al (2009) 2.73 (1.64, 4.55) 2.05
Ji L et al (2009) 0.37 (0.27, 0.50) 3.03
Wang MM et al (2010) 0.93 (0.74, 1.16) 3.41
Jelonek K et al (2010) 1.17 (0.85, 1.61) 2.91
Jelonek K et al (2010) 0.50 (0.35, 0.72) 2.72
Brevik A et al (2010) 0.90 (0.72, 1.11) 3.44
Jelonek K et al (2010) 0.86 (0.59, 1.25) 2.63
Osawa K et al (2010) 1.74 (1.13, 2.68) 2.37
Wang M et al (2010) 1.02 (0.79, 1.32) 3.26
Ye CC et al (2010) 1.06 (0.75, 1.51) 2.77
Kuasne H et al (2011) 1.50 (1.05, 2.15) 2.74
Zhonghua L et al (2011) 1.67 (1.19, 2.33) 2.85
Cao Q et al (2011) 1.18 (1.01, 1.38) 3.70
Huang LZ et al (2011) 1.01 (0.84, 1.21) 3.59
Deng Q et al (2011) 0.80 (0.63, 1.00) 3.40
Cincin Z et al (2012) 2.02 (1.41, 2.88) 2.75
Li Y et al (2013) 1.06 (0.89, 1.26) 3.65
Subtotal (I-squared = 80.7%, p = 0.000) 1.07 (0.97, 1.19) 100.00
.
Overall (I-squared = 80.7%, p = 0.000) 1.07 (0.97, 1.19) 100.00
NOTE: Weights are from random effects analysis
.5
1
4
