# Supplementary material for: APEX Nuclease (Multifunctional DNA Repair Enzyme) 1 Gene Asp148Glu Polymorphism and Cancer Risk: A Meta-Analysis Involving 58 Articles and 48903 Participants
Source: PLoS One. 2013 Dec 12;8(12):e83527. doi: 10.1371/journal.pone.0083527 (PMC3861501; doi:10.1371/journal.pone.0083527)
Supplement: Checklist S1 — MOOSE checklist. (DOC) [file pone.0083527.s008.doc]

**Checklist S1.** MOOSE Checklist

**APEX nuclease (multifunctional DNA repair enzyme) 1 gene Asp148Glu polymorphism and cancer risk: a meta-analysis involving 58 articles and 48903 participants**

Dan Hu^1,2^, Xiandong Lin^1,2^, Hejun Zhang^1,2^, Xiongwei Zheng^1,2^, Wenquan Niu^3^

**Author affiliations:**

^1^Department of Pathology and ^2^Fujian Provincial Key Laboratory of Translational Center Medicine, Fujian Provincial Tumor Hospital, Teaching Hospital of Fujian Medical University, Fuzhou, Fujian province, China; ^3^State Key Laboratory of Medical Genomics, Ruijin Hospital, School of Medicine, Shanghai Jiao Tong University, Shanghai, China.

^#^**Correspondence** to:

Wenquan Niu, Ph.D. Associate Prof. or Xiongwei Zheng, M.D. Ph.D. Prof.

Address: Ruijin Second Road 197, Shanghai 200025, China.

Phone: +86 21 64370045 *ext.* 610905; Fax: +86 21 64333548;

E-mail: niuwenquan_shcn@163.com (WN) or agu1960@126.com (XZ).

| **Criteria** | | **Brief description of how the criteria were handled in the meta-analysis** |
| --- | --- | --- |
| **Reporting of background should include** | |  |
| √ | Problem definition | The results of individual association studies in the literature, however, are often controversial and inconclusive. Taking lung cancer as an example, the *APEX1* 148Glu allele was a risk-conferring factor in Caucasians, but a risk-reducing factor in Asians. As a caveat, this lack of consistency might be attributable to the presence of genetic heterogeneity across ethnic populations, the insufficient sample sizes involved, and the possibly uncontrolled confounding effects. |
| √ | Hypothesis statement | The *APEX1* Asp148Glu polymorphism is associated with the risk of developing cancer. |
| √ | Description of study outcomes | Cancer (all types) |
| √ | Type of exposure or intervention used | *APEX1* Asp148Glu polymorphism (allelic and dominant models) |
| √ | Type of study designs used | Our analyses were limited to the studies that strictly fulfilled the following inclusion criteria (all points must be satisfied for inclusion): (1) clinical endpoint (dependent variable): all types of cancers; (2) study design: either retrospective or nested case-control design; (3) independent variables: the genotype and/or allele counts of the *APEX1* Asp148Glu polymorphism. Studies were excluded (one point was sufficient for exclusion) if they investigated the progression, severity, phenotype modification, and the response to treatment or survival, as well as if they were conference abstracts, case reports or series, editorials, narrative reviews, and the non-English and non-Chinese articles. |
| √ | Study population | We restricted articles published in English and Chinese languages. |
| **Reporting of search strategy should include** | |  |
| √ | Qualifications of searchers | The credentials of the investigator Dan Hu and Wenquan Niu are indicated in the author list. |
| √ | Search strategy, including time period included in the synthesis and keywords | PubMed from 1990 – May 1, 2013  EMBASE from 1990 – May 1, 2013  Wanfang 1995 – May 1, 2013  CNKI 1995 – May 1, 2013 |
| √ | Databases and registries searched | PubMed and EMBASE, Wanfang and CNKI |
| √ | Search software used, name and version, including special features | We did not employ a search software. |
| √ | Use of hand searching | The reference lists of all the retrieved articles as well as those of reviews on the same topic were also searched to identify the additional missing articles. |
| √ | List of citations located and those excluded, including justifications | The citation list is available in the References section. |
| √ | Method of addressing articles published in languages other than English | Searching results were limited to studies with a case-control design and articles published in the English or Chinese language. |
| √ | Method of handling abstracts and unpublished studies | We did not contacted authors for abstracts and unpublished studies on the associations of the *APEX1* Asp148Glu polymorphism with all types of cancers. |
| √ | Description of any contact with authors | We did not contacted authors for abstract and unpublished studies on the associations of the *APEX1* Asp148Glu polymorphism with all types of cancers. |
| **Reporting of methods should include** | |  |
| √ | Description of relevance or appropriateness of studies assembled for assessing the hypothesis to be tested | Detailed inclusion and exclusion criteria were described in the methods section. |
| √ | Rationale for the selection and coding of data | Data extracted from each of the studies were relevant to the population characteristics, study design, and possible effect modifiers of the associations of the *APEX1* Asp148Glu polymorphism with all types of cancers. |
| √ | Assessment of confounding | Subgroup analyses were conducted according to the cancer type, ethnicity of the study populations (Caucasian, Asian, African-American, or mixed), study design (population-based or hospital-based), the total sample size (<300 subjects or ≥300 subjects), and the quality score (score <7 or score ≥7). |
| √ | Assessment of study quality, including blinding of quality assessors; stratification or regression on possible predictors of study results | Overall and subgroup analyses were conducted to explore the associations of the *APEX1* Asp148Glu polymorphism with all types of cancers. |
| √ | Assessment of heterogeneity | Heterogeneity of studies was assessed by the *I*^2^ statistic that provides the relative amount of variance of the summary effect due to the between-study heterogeneity. |
| √ | Description of statistical methods in sufficient detail to be replicated | Description of methods of meta-analyses, subgroup analyses, and assessment of publication bias are detailed in the methods. |
| √ | Provision of appropriate tables and graphics | We included Figure 1 for the flow diagram of search strategy and study selection; Figure 2 for the trim-and-ﬁll funnel plots for studies investigating the effect of the *APEX1* Asp148Glu polymorphism on cancer risk under allelic and dominant models; Table 1 for the baseline characteristics of the study populations analyzed in this meta-analysis; Table 2 for the overall and subgroup estimates of the *APEX1* Asp148Glu polymorphism associations with cancers under allelic and dominant models, and additionally Table S1 for the criteria for quality assessment of genetic associations of APEX1 Asp148Glu polymorphism with cancer risk; Supplementary Table S2 for the genotype distributions and allele frequencies of the APEX1 Asp148Glu polymorphism between cancer patients and controls of all examined populations in this meta-analysis; Supplementary Figure S1 for the forest plots of the lung, bladder, colorectal cancer (the upper panel), and prostate, breast, gastric cancers (the lower panel) in subgroup analyses by cancer type for the *APEX1* Asp148Glu polymorphism under the allelic model; Supplementary Figure S2 for the forest plots of the Caucasians (the upper panel), Asians (the middle panel), African-Americans and mixed populations (the lower panel) in subgroup analyses by ethnicity for the *APEX1* Asp148Glu polymorphism under the allelic model; Supplementary Figure S3 for the forest plots of the hospital-based studies (the upper panel), and population-based studies (the lower panel) in subgroup analyses by study design for the *APEX1* Asp148Glu polymorphism under the allelic model; Supplementary Figure S4 for the forest plots of the small studies (the upper panel), and large studies (the lower panel) in subgroup analyses by sample size for the *APEX1* Asp148Glu polymorphism under the allelic model; Supplementary Figure S5 for the forest plots of the high-quality studies (the upper panel), and low-quality studies (the lower panel) in subgroup analyses by sample size for the *APEX1* Asp148Glu polymorphism under the allelic model. |
| **Reporting of results should include** | |  |
| √ | Graph summarizing individual study estimates and overall estimate | Supplementary Table S2 |
| √ | Table giving descriptive information for each study included | Table 1 |
| √ | Results of sensitivity testing | Table 2, Table 3, and Supplementary Figure S1-S5 |
| √ | Indication of statistical uncertainty of findings | The odds ratios and 95% confidence intervals were presented with all summary estimates, *I*^2^ values and results of sensitivity analyses. |
| **Reporting of discussion should include** | |  |
| √ | Quantitative assessment of bias | Subgroup analyses indicate heterogeneity in strengths of the associations due to most common biases in overall and subgroup analyses. |
| √ | Justification for exclusion | We excluded studies that had the genotype counts deviating from the Hardy-Weinberg equilibrium. |
| √ | Assessment of quality of included studies | Our analyses were limited to the studies that strictly fulfilled the following inclusion criteria (all points must be satisfied for inclusion): (1) clinical endpoint (dependent variable): all types of cancers; (2) study design: either retrospective or nested case-control design; (3) independent variables: the genotype and/or allele counts of the *APEX1* Asp148Glu polymorphism. |
| **Reporting of conclusions should include** | |  |
| √ | Consideration of alternative explanations for observed results | None declared in the Discussion. |
| √ | Generalization of the conclusions | Via a meta-analysis of the data from 58 articles and on 48903 participants, we investigated the association of the non-synonymous polymorphism Asp148Glu in *APEX1* with cancer risk. The principle finding of this study was that the *APEX1* 148Glu allele was associated with the significant risk of developing gastric cancer, even after the Bonferroni correction. Moreover, our subgroup analyses indicated that ethnicity might be an underlying cause of heterogeneity between studies. Although other sources of heterogeneity cannot be easily ruled out, this study, to the best of our knowledge, is so far the largest meta-analysis examining the association of the *APEX1* Asp148Glu polymorphism with cancer risk. |
| √ | Guidelines for future research | For practical reasons, we hope that this study will not remain just another endpoint of research instead of a beginning to establish the background data to understand the roles of the *APEX1* in carcinogenesis. |
| √ | Disclosure of funding source | This study was supported by the Natural Science Foundation of Fujian Province (2012J01328). |
